# Contrasting cryptofaunal responses to seabird nutrient inputs illuminate coral reef productivity pathways

**DOI:** 10.1002/ecy.70453

**Published:** 2026-07-22

**Authors:** Laura‐Li Jeannot, Ruth E. Dunn, M. Joyce P. Velos, Gareth J. Williams, Cassandra E. Benkwitt, Nicholas A. J. Graham, Simon J. Brandl

**Affiliations:** ^1^ Lancaster Environment Centre Lancaster University Lancaster UK; ^2^ Centre d'Ecologie Fonctionnelle et Evolutive Montpellier France; ^3^ Department of Marine Science, Marine Science Institute The University of Texas at Austin Port Aransas Texas USA; ^4^ School of Ocean Sciences Bangor University Menai Bridge, Anglesey UK

**Keywords:** coral reef ecology, cryptobenthic fishes, cryptofauna, nutrient enrichment, nutrient subsidies, reef invertebrates, seabird guano, stable isotope analysis

## Abstract

Nutrients moving across ecosystems can propagate through food webs via distinct trophic pathways. Their integration into food webs is often mediated by small, abundant taxa that channel subsidies from primary producers to higher trophic levels. On coral reefs, cryptobenthic fishes and invertebrates can fill these roles, yet their responses to allochthonous nutrients are poorly understood. We compared cryptofaunal communities and associated trophic dynamics across nutrient‐rich islands with intact seabird populations and nutrient‐poor rat‐infested islands in the Chagos Archipelago, Indian Ocean. Specifically, we evaluated how the presence of seabird colonies influenced cryptofaunal nutrient assimilation, biomass, structure, and isotopic niche dynamics, and how these shifts propagated to mesopredators and higher order piscivorous and invertivorous fishes. Most cryptobenthic fishes and some invertebrates showed nutrient enrichment near seabird colonies. Although the isotopic niches of cryptobenthic fishes and invertebrates overlapped, niche widths diverged in nutrient‐rich environments, with invertebrates exhibiting broader niches and cryptobenthic fishes remaining comparatively constrained, reflecting differential access to shared resources. Mixing models also reflected a shift toward cryptobenthic fishes in the diet of a mixed carnivore near seabird colonies. Community patterns revealed strong asymmetries: Nutrient‐rich reefs supported twice the amount of cryptobenthic fish biomass and a 10‐fold increase in larger piscivorous fish productivity, while reefs near rat‐infested islands were characterized by relatively greater cryptic invertebrate biomass and higher invertivore productivity. Taken together, these results suggest that cryptobenthic fishes' capacity to capitalize on seabird nutrients may allow them to exert competitive dominance and top‐down control over invertebrates in nutrient‐rich environments, thereby amplifying piscivorous pathways, while nutrient‐poor environments appear to favor invertebrates and their consumers. Rather than reflecting uniform biomass loss under rat invasion, these patterns indicate a shift in trophic routing from fish‐mediated to invertebrate‐mediated energy channels. Our findings demonstrate that cross‐ecosystem nutrient vectors do not simply enhance reef productivity but reorganize trophic structure. Therefore, invasive species that sever these linkages can drive the emergence of alternative food‐web configurations with distinct energetic pathways.

## INTRODUCTION

Nutrient flow, both across ecosystems and through food webs, is critical for sustaining productivity, especially for oligotrophic ecosystems. On nutrient‐limited coral reefs that surround isolated atolls and islands, seabirds act as key cross‐ecosystem nutrient vectors, feeding in the open ocean and depositing nitrogen‐ and phosphorus‐rich guano where they roost and nest (Benkwitt et al., 2022; Gove et al., 2016; Graham et al., 2018; Jones et al., 2025; McCauley et al., 2012; Otero et al., 2018). In doing so, they introduce oceanic nutrients to otherwise nutrient‐poor island systems, which leach into adjacent marine environments, fertilizing reefs and boosting productivity across trophic levels, and underscoring the central role seabird‐derived nutrients play in structuring reef ecosystems (Benkwitt et al., 2023; Benkwitt, Gunn, et al., 2021). However, invasive rats (*Rattus* spp.) have extirpated seabird colonies from many islands, effectively severing the ocean‐to‐island‐to‐reef nutrient pipeline and causing a marked reduction in nutrient export to surrounding reefs (Jones et al., 2008).

Bottom‐up enrichment can propagate through food webs via multiple trophic pathways (Dunn et al., 2025), yet the mechanisms through which added nutrients are funneled up food webs and influence fish biomass and productivity are only partially understood. As a result of primary producer nutrient enrichment, larger herbivorous reef fishes (e.g., parrotfishes and surgeonfishes) show elevated growth and biomass associated with seabird presence (Benkwitt, Taylor, et al., 2021; Benkwitt, Zora, et al., 2025; Graham et al., 2018). Higher level consumers also respond to nutrient‐driven changes, as reefs with seabird nutrient input support greater biomass of carnivorous fishes (Benkwitt et al., 2019; Graham et al., 2018). In contrast, little is known about how nutrient availability affects carnivores' underlying prey communities, and the specific trophic routes by which seabird‐derived nutrients propagate through reef food webs. A primary mechanism for the integration of seabird nutrients is likely through the benthic community, where otherwise nutrient‐limited primary producers (e.g., turf algae) take up seabird‐derived nutrients (Benkwitt, Zora, et al., 2025; Graham et al., 2018). Organisms strongly associated with the benthos that derive energy from its primary producers may therefore act as critical vectors in the transfer of seabird nutrients to higher trophic levels. These include cryptobenthic fishes (e.g., gobies, blennies, triplefins) and small motile invertebrates (e.g., crustaceans, molluscs, worms sensu lato), which collectively dominate reef biodiversity and biomass (Glynn & Enochs, 2011; Plaisance et al., 2021).

The role of small consumers in nutrient and energy fluxes exhibits strong environmental context specificity and may therefore differ between nutrient‐rich and nutrient‐poor habitats (Brandl et al., 2025). Evidence suggests that, in isolation, both cryptofaunal guilds respond to seabird‐derived nutrient input, with elevated δ^15^N near seabird colonies indicative of seabird‐nutrient uptake, and larger sizes in nutrient‐rich environments (Appoo et al., 2023, 2024; Healing et al., 2024; Jeannot et al., 2025). Yet, responses to nutrient‐rich conditions may not be uniform: While seabird‐subsidized systems may favor taxa with higher rates of nutrient uptake and elevated metabolic demands, along with their predators, others can emerge as dominant in nutrient‐depleted environments such as those influenced by invasive species (e.g., rats) (Pearson et al., 2022). Contrasting nutrient regimes may therefore foster diverging ecosystem configurations, trophic pathways, and food‐web structures. For small benthic consumers, the overlap between the habitats they occupy and the resources they use (e.g., detritus, benthic primary producers) implies that any competitive advantage under enriched or depleted conditions, such as enhanced assimilation efficiency, faster growth, or greater trophic flexibility, could shift patterns of resource partitioning between guilds. Such dynamics may be reflected in changes to isotopic niches, which provide insight into how nutrient availability shapes resource use and potential competitive interactions (Abbey‐Lee et al., 2013). Moreover, several cryptobenthic fishes prey directly on invertebrates (e.g., dottybacks), further reinforcing potential interguild asymmetry in different nutrient environments, and influencing how energy is routed from lower trophic levels to mesopredators and larger piscivorous and invertivorous consumers (Ashworth et al., 2014). How piscivory, invertivory, and overall reef productivity respond to nutrient variation is therefore central to determining whether invasive species simply reduce nutrient supply or instead drive a reorganization of trophic pathways and energy routing within reef ecosystems.

Here, we compare the uptake of environmentally available nutrients in cryptobenthic reef fishes and cryptic invertebrate communities and evaluate how they may influence associated piscivorous and invertivorous trophic pathways. We focus on a remote reef system, the Chagos Archipelago in the Indian Ocean under high (rat‐free, seabird‐rich islands) versus low (rat‐infested, seabird‐poor islands) nutrient conditions, and we address four questions across hierarchical levels of ecological organization, beginning with nutrient assimilation and community responses within cryptofaunal guilds before scaling up to trophic interactions and food‐web consequences. First, considering nutrient uptake and community responses of cryptobenthic fishes and invertebrates separately, we ask: (1) Do fish and invertebrate cryptofaunal communities assimilate seabird‐derived nutrients? (2) How does seabird‐nutrient uptake affect cryptobenthic fish and invertebrate community biomass, abundance, and structure? We then examine how nutrient enrichment mediates competitive and predatory interactions within and beyond these guilds: (3) Does nutrient enrichment alter isotopic niches of cryptobenthic fishes and invertebrates and their potential competitive interactions? (4) How do shifts in cryptofauna influence mesopredator resource use and productivity of piscivores and invertivores?

## METHODS

### Field sampling

**FIGURE 1:**
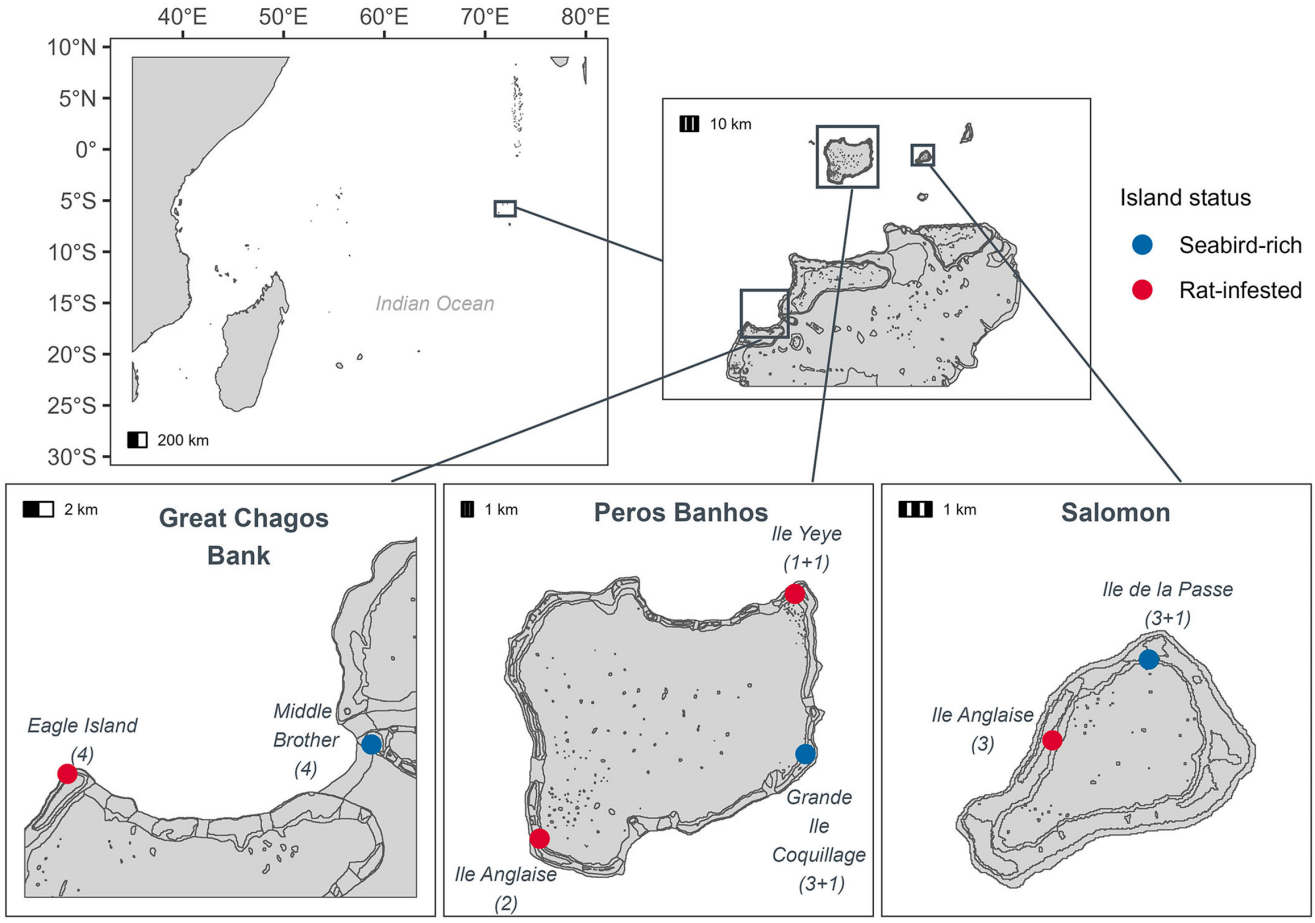
Map of the sampling locations. Numbers within parentheses represent the number of sampling stations per island, and +1 indicates that samples from an additional sampling station were used for stable isotope analysis only. See Appendix S1: Table S1 for further details about sampling sites (location, seabird density, curved surface length of sampled area).

This study was conducted across the remote atolls of the Chagos Archipelago, Indian Ocean, a large, isolated and largely uninhabited marine protected area with no direct human impacts on the studied atolls. Despite being a globally important area for seabird populations (Carr et al., 2023), the presence of invasive predatory black rats (*Rattus rattus*) has significantly depleted seabird populations on some islands where rats were introduced several hundred years ago. Seabird density is therefore approximately 760 times greater on rat‐free islands, with approximately 250 times more seabird‐derived nitrogen fertilizing the reef surrounding these islands and promoting the growth of corals, herbivorous damselfish, and parrotfish (Benkwitt et al., 2023; Benkwitt, Taylor, et al., 2021; Graham et al., 2018). We used these differences in seabird‐derived nutrient subsidies between islands with and without rats to test how nutrient inputs from seabirds affect cryptofauna. We collected cryptobenthic fishes and cryptic mobile invertebrates from 17 to 31 October 2023 from seven islands (three seabird‐rich and four seabird‐poor) spanning three atolls: Salomon, Peros Banhos, and the Great Chagos Bank (Figure 1). All cryptofauna was collected on the lagoonal side of the islands except for the rat‐infested Eagle Island (Great Chagos Bank) where environmental conditions in the lagoon prohibited sampling. All individuals were caught on SCUBA at an average depth of 5.9 ± 1.9 m, in reef crest and back reef slope habitats within 150 m from islands.

Each sampling station consisted of a coral outcrop selected to be similar in shape, isolation, and size (150–260 cm curved surface length; 1.4–4.3 m^2^ surface area) and sampling suitability for cryptobenthic fish and invertebrate sampling. Sampling followed well‐established cryptofauna collection methods (Ackerman & Bellwood, 2002; Brandl et al., 2018; Stier & Leray, 2014) and is further detailed in Appendix S1: Section S1. In total, cryptobenthic fishes and invertebrates were sampled across 23 sampling stations (seabird‐rich location: 12 stations; seabird‐poor location: 11 stations; *n* = 2–4 per island; Appendix S1: Table S1). To compare visually conspicuous piscivorous and invertivorous fish communities, underwater visual censuses (UVC) were conducted both in 2021 and in 2023 following Benkwitt et al. (2019) and Graham et al. (2018).

### Isotope analyses

Individuals of seven cryptobenthic fish species—including six gobies and one carnivorous dottyback (*Chlidichthys chagosensis*)—and seven cryptic invertebrate taxa were selected for stable isotope analysis due to high abundance and presence at both seabird‐rich and seabird‐poor islands (Appendix S1: Table S2). For fish, nitrogen and carbon isotope composition were measured concurrently. For invertebrate carbon isotope composition, samples were weighed, sealed into silver capsules, and acid‐fumigated for 24 h with concentrated HCl vapor in a desiccator for 24 h to remove inorganic carbon, and were thoroughly oven‐dried for 48 h prior to analysis and sealed. Thereafter, carbon and nitrogen isotope compositions were measured at The University of Texas at Austin (Marine Science Institute, Core Isotope Facility in Port Aransas, Texas, USA). Further stable isotope analysis details, including lipid normalization for invertebrate δ^13^C, are presented in Appendix S1: Section S2.

### Data analyses

To explore δ^15^N across different taxa as a function of island status (fixed effect, two levels: seabird‐rich and rat‐infested) and island (nested within atoll as a random effect to account for local variation in δ^15^N), we fit Gaussian Bayesian linear mixed‐effects models. Separate models were first fit for each guild, and interactions with invertebrate taxa and cryptobenthic fish species, respectively, were then included to investigate taxonomic level influence on δ^15^N. We further explored the interaction between island status and cryptobenthic fish or invertebrate length on δ^15^N to investigate differential enrichment in larger, higher metabolism individuals. Length was log‐transformed and centered to account for allometric scaling and to reduce collinearity between length and its interaction with island status. Sampling depth and reef habitat were similar within each atoll and were therefore accounted for using this model structure.

We compared the density and biomass of cryptic invertebrate and cryptobenthic fishes across seabird‐rich and rat‐infested islands, standardized by curved surface length‐derived sampling area, using log‐link Gamma Bayesian linear mixed‐effects models. Three stations were characterized by high‐swell conditions that affected sampling and were therefore removed from community analyses due to resulting concerns about the comprehensiveness of the collection, resulting in a total of 20 stations (seabird‐rich location: 10 stations; rat‐infested location: 10 stations; Appendix S1: Table S1) for this analysis.

To explore the effect of seabirds on the coral outcrop‐level ratio of cryptobenthic fishes to invertebrates, two lognormally distributed Bayesian mixed‐effects models were run with ratios of fish to invertebrate density and biomass as response variables, and island status and island within atoll as fixed and random effects, respectively. Effects on community composition were further investigated using a redundancy analysis (RDA) with island status and atoll as constraints. A SIMPER (Similarity Percentage) analysis based on abundance using the vegan package (Oksanen et al., 2001) was used to identify taxa primarily responsible for differences between rat‐infested and seabird‐rich islands.

To test for potential competitive interactions between fishes and invertebrates, isotopic niches were investigated using the SIBER package (Jackson et al., 2011). We fitted ellipses using JAGS, computed percent overlap, and calculated the standard ellipse area corrected for small samples (SEAc) for both cryptobenthic fish and invertebrate communities across seabird‐rich and rat‐infested islands. Greater isotopic niche overlap reflects increased similarity in trophic resource use and, when coupled with shifts in niche width, may indicate altered competitive dynamics. Larger SEAc indicates broader trophic niches and more diverse resource use, while smaller areas indicate more constrained or specialized resource use.

To investigate whether seabird nutrients influenced the proportion of fish or invertebrates consumed by a cryptobenthic mesopredator, we estimated the reliance of the dottyback *C. chagosensis* on either source. This was done using a two‐source dual‐biotracer (δ^15^N and δ^13^C) isotope mixing models with the R package MixSIAR (Stock et al., 2018). Only >30 mm *C. chagosensis* were selected due to the high reliance of smaller individuals on δ^13^C‐enriched resources (i.e., planktonic resources) (Appendix S1: Figure S1). Prey sources were other cryptobenthic fishes and cryptic invertebrates. We chose a trophic discrimination factor of 0.4‰ ± 1.3 SD for δ^13^C which is considered widely applicable in aquatic food webs (Post, 2002). As predator size is expected to influence prey uptake, *C. chagosensis*' total length was used as a continuous variable.

Finally, to investigate potential biomass and productivity differences in larger reef fish communities, larger fish recorded from UVC data were partitioned into feeding groups (Sandin & Williams, 2010) and biomass was calculated using length–mass relationships from FishBase Bayesian estimators (Froese et al., 2014; Froese & Pauly, 2000). We computed yearly productivity based on Benkwitt et al. (2020), Brandl et al. (2019), and Morais and Bellwood (2020). Biomass and productivity at each transect were integrated in log‐link Gamma Bayesian models to evaluate the effect of seabird nutrients, with island within atoll and year as random effects. Model information, prior specifications, and details regarding chains and convergence are presented in Appendix S1: Section S3 and Table S3.

## RESULTS

Seven cryptobenthic fish families were found across the three atolls (Gobiidae, Tripterygiidae, Blenniidae, Syngnathidae, Pseudochromidae, Apogonidae, and Gobiesocidae), with 702 fish across 64 species from the 20 stations that were retained for community analyses. For invertebrates, we found 618 individuals across 35 taxa (Appendix S1: Table S4).

### Seabird‐nutrient assimilation by cryptofauna

**FIGURE 2:**
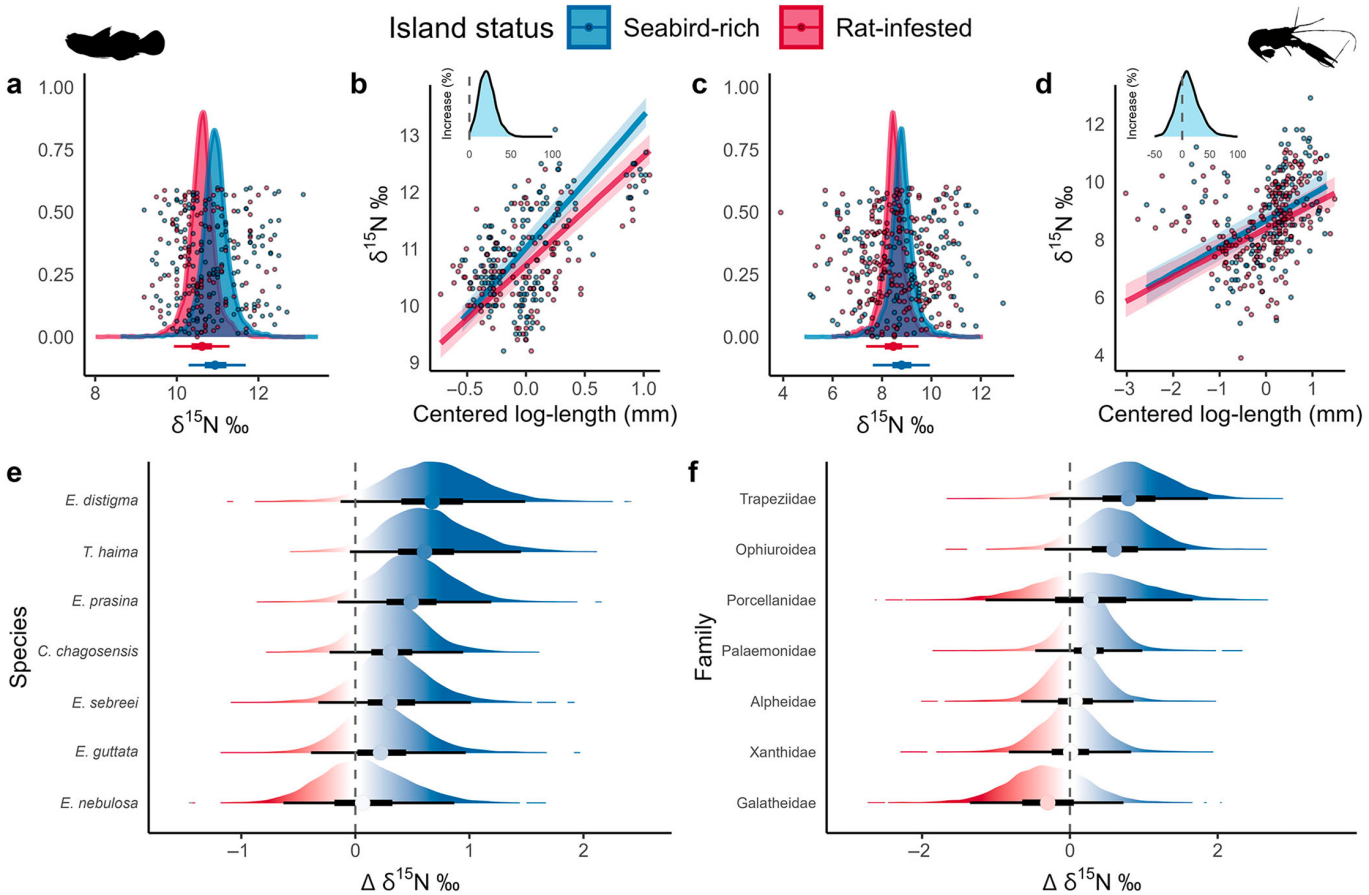
Effects of seabird presence on cryptofauna δ^15^N. Density curves and caterpillar plots show δ^15^N distributions for cryptobenthic (a) fish and (c) invertebrate guilds, grouped by seabird presence. Caterpillar plots represent fitted values from Bayesian linear models, with 50% (bold lines) and 95% (thin lines) credible intervals based on 1000 posterior draws. Relationship between δ^15^N and length for (b) fish and (d) invertebrates, grouped by seabird presence. Shaded bands represent 50% credible intervals, and individual dots correspond to raw data points. Inset plots represent posterior draws of the percentage of slope increase near seabird islands. Contrasts between seabird‐rich and rat‐infested islands by cryptobenthic (e) fish species and (f) invertebrate taxa. Fish and invertebrate silhouettes created by Simon J. Brandl and Laura‐Li Jeannot.

Both cryptobenthic fish (Figure 2a) and invertebrates (Figure 2c) showed moderately high evidence of nutrient enrichment near seabird islands (92% and 81% posterior probability of higher δ^15^N near seabird islands, respectively), with significant variability at finer taxonomic resolution within each group. Within cryptobenthic fishes, posterior probabilities of higher δ^15^N ranged from 77% to 96%, with only *Eviota nebulosa* presenting no clear effect (56% posterior probability; Figure 2e; Appendix S1: Table S5). Three invertebrate taxa also showed moderate to strong evidence (>75%) of higher δ^15^N near seabird islands: trapeziid (93% posterior probability) and porcellanid (80% posterior probability) crabs, and brittle stars (ophiuroids) (90% posterior probability), while all others ranged from 28% (galatheid squat lobsters) to 66% (palaemonid shrimps) posterior probabilities (Figure 2f).

Body size influenced δ^15^N enrichment differently according to guild: Seabird presence had a positive effect on this relationship for cryptobenthic fishes, with a 20.7% stronger correlation between length and δ^15^N for islands with seabirds, suggesting that larger cryptobenthic fishes with higher metabolic demand display higher enrichment in δ^15^N near seabird islands (>99% posterior probability of positive effect of seabird presence on the correlation between length and δ^15^N; Figure 2b). No such overall trend was detected in invertebrates, where there was little to no observable effect of seabird presence on the strength of the correlation between δ^15^N and length (69% posterior probability; Figure 2d), suggesting larger size and metabolic demand correlates with higher δ^15^N assimilation near seabird islands for cryptobenthic fishes only.

### Cryptofaunal biomass, density, and community composition

**FIGURE 3:**
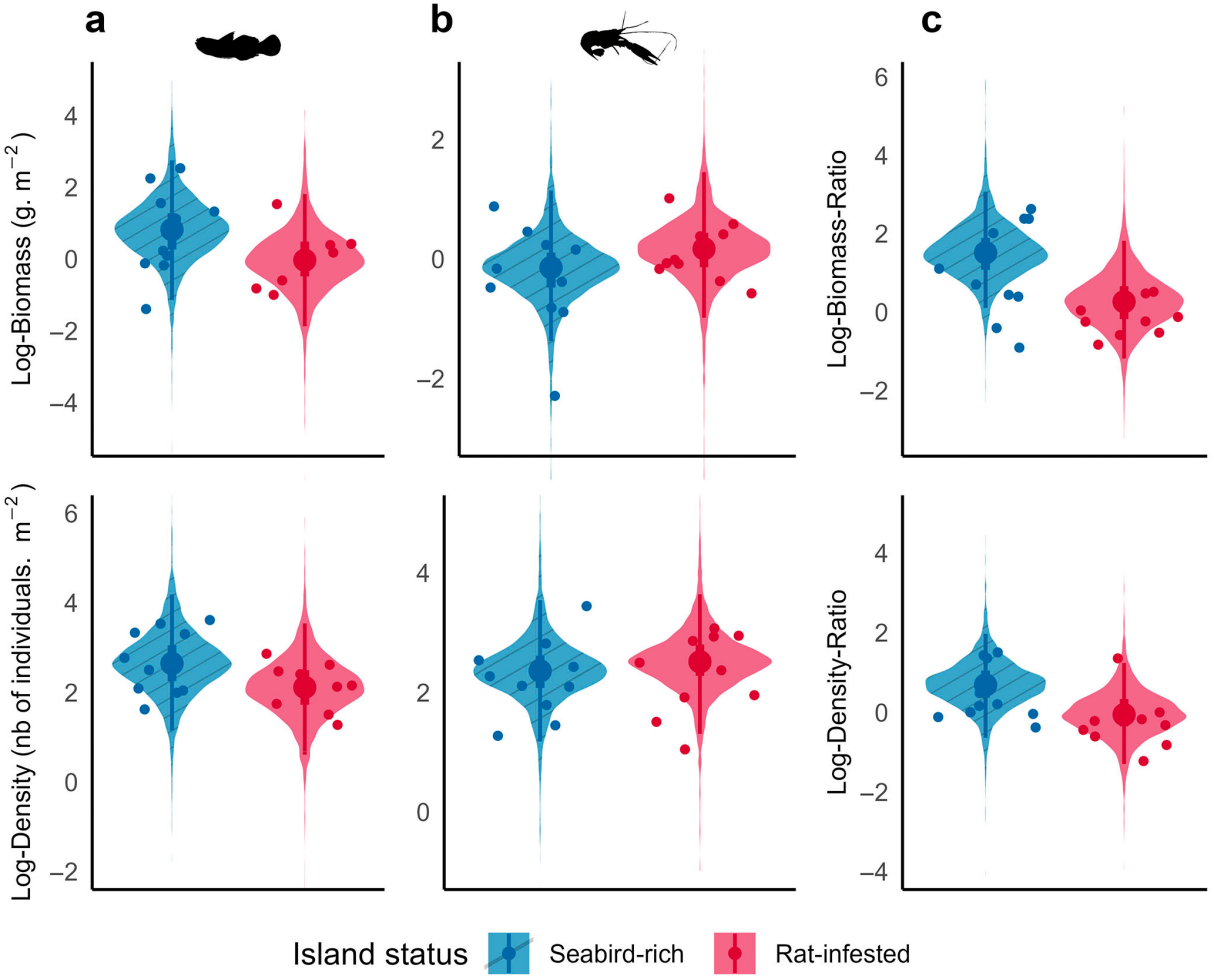
Posterior predictive distributions for (a) cryptobenthic fish biomass and density, (b) cryptic invertebrate biomass and density, and (c) fish to invertebrate ratios. Points represent median estimates, and lines represent 95% and 50% highest posterior density intervals. Fish and invertebrate silhouettes created by Simon J. Brandl and Laura‐Li Jeannot.

There was strong evidence of higher cryptobenthic fish biomass near seabird islands (96% posterior probability) and moderate evidence of higher density (84% posterior probability). Estimated cryptobenthic fish biomass was 3.91 g/m^2^ (95% HDI: [0.01; 11.66]) near seabird islands, more than 2.2 times the estimated 1.76 g/m^2^ (95% HDI: [0.01; 4.33]) near rat‐infested islands (Figure 3a). This increase was mostly driven by gobies and triplefins (Appendix S1: Figures S2 and S3). A weaker but opposite pattern emerged for invertebrates, which had a higher likelihood of greater or equal biomass and density near rat‐infested islands (81% and 66% posterior probability, respectively; Figure 3b). Ophiuroid, and trapeziid and porcellanid crab biomasses were lowest near seabird islands (Appendix S1: Figure S4). Likewise, in terms of relative biomass and abundance, the ratio of cryptobenthic fishes to invertebrates was consistently higher near seabird islands (97% posterior probability of higher biomass), with an average ratio of cryptobenthic fish biomass to invertebrate biomass of 0.9:1 near rat‐infested islands to 5.2:1 near seabird‐rich islands, indicating differences in cryptofauna community composition according to island status (Figure 3c).

Both cryptobenthic fish and invertebrate communities differed across the two treatments. There was species‐level compositional dissimilarity (redundancy analysis: *p*‐value_Island status_ = 0.047, *p*‐value_Atoll_ = 0.005, *R* ^2^_adj_ = 0.171) for cryptobenthic fishes, and taxa‐level dissimilarity (redundancy analysis: *p*‐value_Island status_ = 0.015, *p*‐value_Atoll_ = 0.132, *R* ^2^_adj_ = 0.129) for cryptic invertebrates. Nine cryptobenthic fish species, including five goby species, significantly contributed to the dissimilarities between treatments (Appendix S1: Table S6) and were more abundant near seabird‐rich islands. Three cryptic invertebrate taxa, including trapeziid and porcellanid crabs, were all consistently more abundant near rat‐infested islands (Appendix S1: Table S7).

### Cryptofaunal isotopic niche variation

**FIGURE 4:**
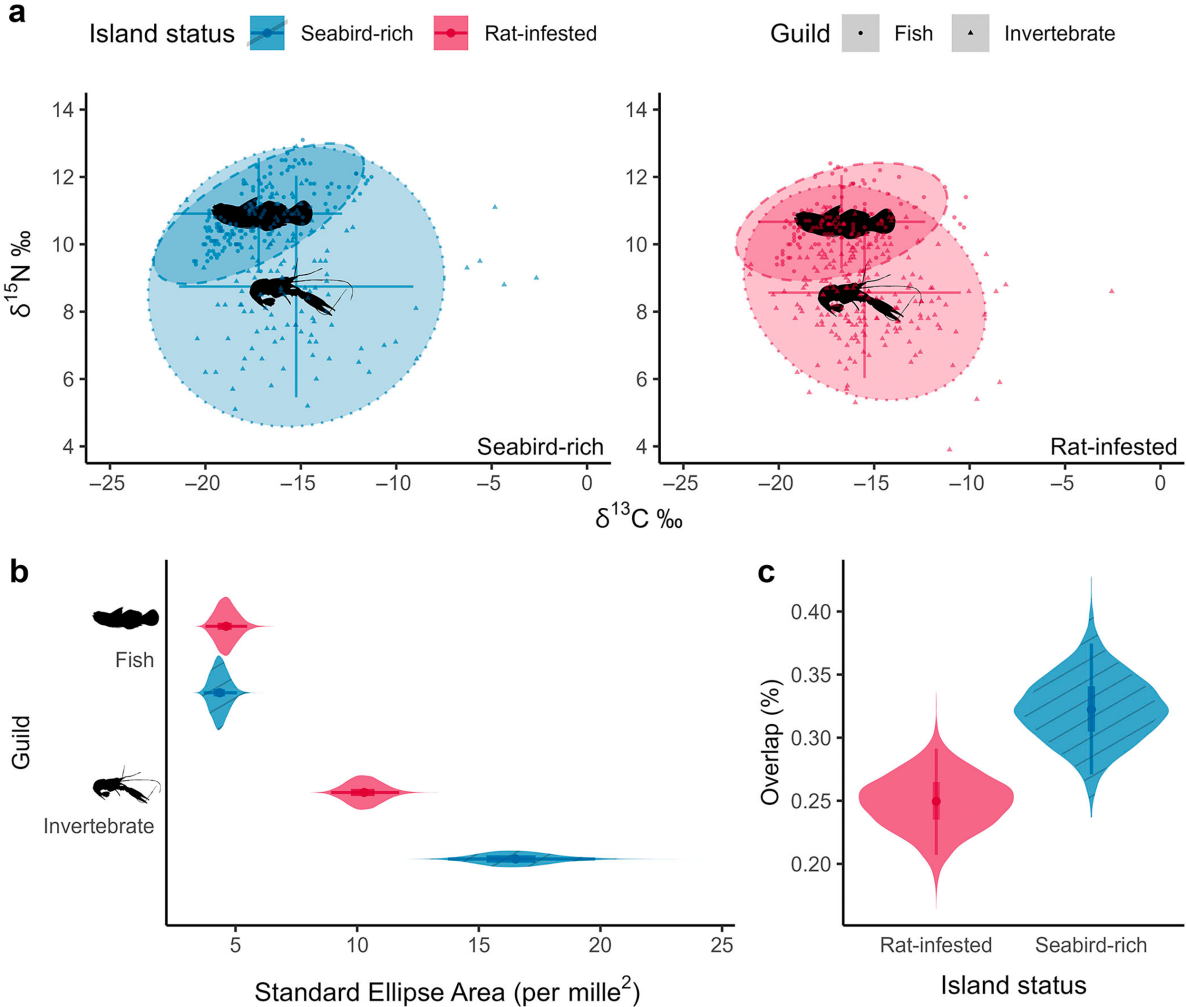
(a) Isotopic biplots of δ^13^C and δ^15^N for cryptobenthic fish and invertebrates grouped by seabird presence. Error bars represent SD, and ellipses are drawn using the normal distribution at a 95% CI. (b) Posterior distributions of Bayesian standard ellipse areas (per mille squared), estimated using posterior draws from a multivariate normal distribution using the SIBER package. (c) Posterior distributions of percentage overlap between cryptobenthic fish and invertebrate isotopic niches based on 4000 draws. Fish and invertebrate silhouettes created by Simon J. Brandl and Laura‐Li Jeannot.

The isotopic niches between cryptobenthic fishes and cryptic invertebrates overlapped regardless of island status. However, the proportional overlap differed according to treatment (98.4% overlap near seabird‐rich islands; 72.8% overlap near rat‐infested islands; Figure 4c). This was due to the corrected standard ellipse area (SEAc) of cryptobenthic fishes shrinking by 6.5% and the isotopic niche of cryptic invertebrates expanding by 61.4% near seabird‐rich islands (Figure 4a,b; Appendix S1: Table S8).

### Higher trophic level biomass and productivity

The influence of seabird presence on higher trophic levels' resource use was illustrated by the cryptobenthic mesopredatory fish *Chlidichthys chagosensis*, which was found in the same coral outcrops as the other cryptobenthic fishes and cryptobenthic invertebrates. *C. chagosensis* fed predominantly on invertebrate food sources irrespective of rat presence, yet relied more on fish as a resource near seabird islands (7.8% instead of 3.7%). This shift was related to total length, with larger individuals relying increasingly on fish (up to 19.4% of total diet by maximum total length measured near seabird islands; up to 9.9% near rat‐infested islands; Appendix S1: Figure S5).

**FIGURE 5:**
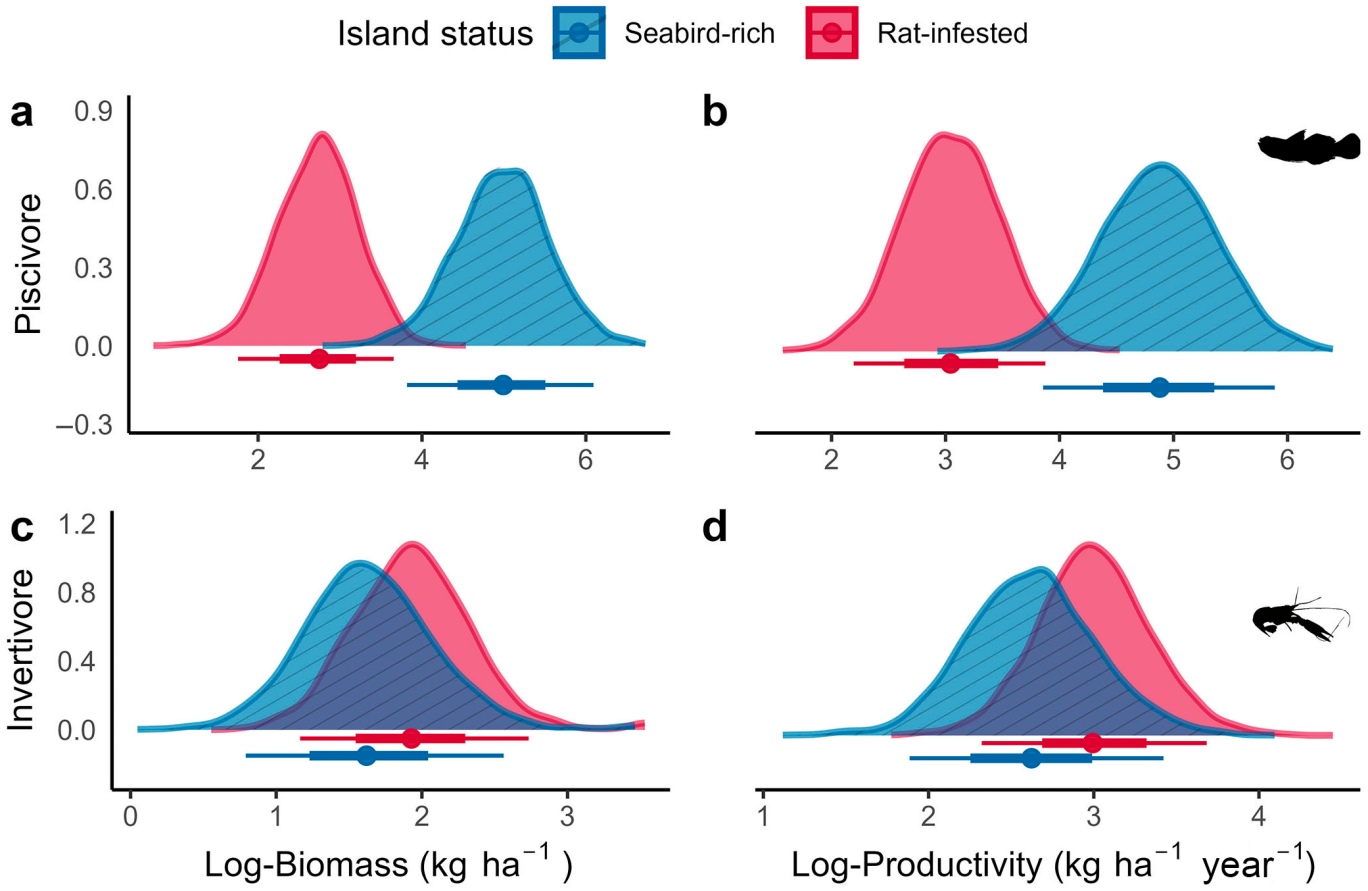
Posterior distributions of (a) biomass and (b) productivity of visually conspicuous piscivorous fishes and (c) biomass and (d) productivity of visually conspicuous invertivorous fishes. Density curves and caterpillar plots (50% and 95% credible intervals) represent fitted values from Bayesian linear models based on 1000 posterior draws. Fish and invertebrate silhouettes created by Simon J. Brandl and Laura‐Li Jeannot.

Posterior estimates revealed substantial increases in both biomass and productivity of visually conspicuous piscivorous fishes near seabird‐rich islands (>99% posterior probability). Piscivorous fish biomass and productivity were enhanced by 82% and 57%, respectively, on the log scale (Figure 5a,b), with an estimated biomass of 170.14 kg/ha (95% HDI: [31.04; 358.85]) near seabird‐rich islands, corresponding to a 10‐fold increase compared to 17.53 kg/ha (95% HDI: [4.28; 36.00]) near rat‐infested islands. In contrast, there was moderate evidence (82% and 85% posterior probability, respectively) that invertivorous fishes exhibited a reduction in both biomass and productivity near seabird islands (18% and 12% lower, respectively, on the log scale) (Figure 5c,d).

## DISCUSSION

Allochthonous nutrient inputs can influence the trophic pathways that sustain consumer communities within an ecosystem. Highly productive, ecologically important, yet often overlooked, benthic cryptofauna represent a cornerstone of energy transfer on coral reefs (Brandl et al., 2018, 2019) and likely play a key role in integrating and transferring seabird nutrients through food webs (Goberdhan et al., 2024; Jeannot et al., 2025; Plaisance et al., 2021; Stella et al., 2011). Despite co‐occurring in up to two‐thirds of reef habitats (Takada et al., 2007), cryptobenthic fishes and invertebrates have rarely been studied together. Here, we investigated how seabird‐derived nutrient subsidies influence coral reef cryptofauna through whole‐community collections of cryptobenthic fishes and cryptic invertebrates. Stable isotope analyses revealed assimilation of seabird‐derived nitrogen by several cryptofaunal consumers. Yet, cryptobenthic fishes and invertebrates exhibited divergent trends in biomass and community structure: Fish populations and biomass increased under nutrient enrichment, whereas motile invertebrate abundance and biomass showed weak and opposite trends. This divergence suggests that bottom‐up fertilization by seabirds does not uniformly elevate all components of the food web, but instead triggers complex interactions likely modulated by life history characteristics, feeding strategies, and predator–prey dynamics. Isotopic niche analyses further revealed divergent trophic responses, as invertebrates expanded their niche space in nutrient‐rich environments while cryptobenthic fishes retained comparatively constrained niches despite substantial overlap, suggesting differential access to shared resources. In turn, as cryptofaunal interactions mediate uptake and trophic transfer to larger fishes, this asymmetry may propagate up the food web: Larger nominal piscivores exhibited stark increases in biomass and productivity in the presence of nutrient enrichment, contrary to nominal invertivores. Collectively, these results suggest that seabird‐vectored nutrients enhance reef productivity by boosting the abundance and biomass of cryptobenthic fishes, which in turn act as efficient conduits that shuttle energy to higher trophic levels that are adapted to feeding on fish prey, whereas nutrient‐poor environments may instead support invertebrate‐mediated pathways and alternative trophic configurations (Appendix S2: Figure S1).

### Contrasting assimilation of seabird‐derived nutrients by cryptofauna

Seabird‐derived nutrients are assimilated into the coral reef cryptofaunal food web, but this appears to depend strongly on consumer identity. On islands with abundant seabird colonies and high guano input, several cryptobenthic fish species and invertebrate taxa had markedly elevated δ^15^N values relative to those from seabird‐depleted islands. This is consistent with previous studies documenting seabird‐nutrient uptake across diverse aquatic invertebrates (e.g., isopods, amphipods, gastropods, decapods; Appoo et al., 2024; Dunn et al., 2025; Healing et al., 2024; Kolb et al., 2010) and cryptobenthic fish assemblages (Jeannot et al., 2025). Here, isotopic enrichment was observed in several *Eviota* species, the goby *Trimma haima*, and cryptobenthic invertebrates (Ophiuroidea, Trapeziidae, Porcellanidae). However, elevated δ^15^N is not always synonymous with the presence or absence of seabird bottom‐up effects (Benkwitt, Taylor, et al., 2021). Similarly, despite evidence for cryptofauna‐wide enrichment, we found inconsistent δ^15^N signals among cryptobenthic fish and invertebrate taxa, echoing findings on larger, visually conspicuous fishes elsewhere (Benkwitt, Bistolas, et al., 2025). Seabird‐nutrient assimilation therefore appears to be mediated by a combination of traits and spatial dynamics that result in heterogeneous uptake across taxa.

The heterogeneity in nutrient uptake among taxa likely reflects both dietary differences and fine‐scale interactions between species' diets and their nutritional landscape, with structurally complex habitats such as coral crevices serving as retention sites. Hydrodynamic conditions may influence how nutrients accumulate locally: Rapidly flushing reefs may homogenize nearshore nutrient landscapes and result in uniform δ^15^N enhancement elsewhere (i.e., fringing reefs, Jeannot et al., 2025). On the other hand, areas with longer residence times and limited mixing such as the lagoons of the Chagos Archipelago (Rayner & Drew, 1984) may amplify spatial heterogeneity and create nutrient micro‐hotspots that exacerbate species and higher taxa‐level divergences. Indeed, peak seabird‐nutrient enrichment occurs in low‐flow shallow areas due to localized seafloor curvature, where organisms that inhabit complex microhabitats (i.e., biofilms and filter feeders) can then sequester dissolved nutrients (Stuart et al., 2025). In line with this, the grazing of the epilithic algal matrix and its detrital aggregates can be an important seabird‐nutrient incorporation pathway for intertidal cryptobenthic fishes and invertebrates (Andrades et al., 2024). Cryptofauna are uniquely positioned to exploit these spatially constrained resources as their small body sizes and microhabitat specialization allow access to nutrient hotspots inaccessible to larger consumers. Enrichment was indeed strongest for species restricted to microhabitats where seabird‐subsidized primary producers or detritus may accumulate, such as crevices (e.g., *E. distigma*, *E. prasina*, Ophiuroidea; Herler, 2007, Stöhr et al., 2012).

Several lines of evidence suggest that seabird‐derived nutrients boost cryptobenthic fish communities more than invertebrates across our study system. The stronger correlation between δ^15^N and cryptobenthic fish total length near seabird islands implies two potential, non‐mutually exclusive scenarios: (1) as fishes grow, they accumulate greater amounts of seabird‐derived nutrients; and (2) fishes grow faster near seabird islands, as previously demonstrated in two larger, non‐cryptobenthic fish species (Benkwitt, Taylor, et al., 2021; Graham et al., 2018). In addition, *E. distigma*, which presented the strongest δ^15^N responses to seabird presence, was also associated with the most significant biomass enhancement near seabird islands. On the other hand, trapeziids and ophiuroids, which also exhibited higher δ^15^N, both showed significantly lower biomass near seabird islands. These patterns hold true at the guild level, with higher biomass of cryptobenthic fishes and a fivefold increase in the proportion of cryptobenthic fish biomass to cryptic invertebrate biomass near seabird islands. This suggests that, while some invertebrates ingest seabird‐derived nutrients, lower biomass near seabird islands may reflect either limited conversion into population‐level biomass or stronger top‐down control from predators, in contrast to the positive biomass response observed in some cryptobenthic fishes.

### Interguild competition and predation

Fishes and invertebrates' ecological responses are intricately linked and, in some cases, interdependent, with some species of gobies, alpheid shrimps, and, in a rare case, a porcellanid crab forming unique symbioses (Werding et al., 2016). Yet, our results are consistent with other studies demonstrating divergent responses across multiple ecosystems (Jackson & Harvey, 1993; Lin et al., 2021; Wyżga et al., 2014), with competition and predation often driving these responses. Our findings provide further evidence that both competitive interactions and top‐down forces may modulate the bottom‐up effects of nutrient enrichment in the cryptofaunal community.

Competition is strongest in horizontal communities made up of sympatric, ecologically similar, and comparably sized individuals. Here, variation in isotopic niche overlap and widths suggests that interguild competition drives access to resources. Greater levels of competition and resource scarcity, as would be observed in the nitrogen‐ and phosphorus‐limited waters near rat‐infested islands, can result in diet specialization (Andrades et al., 2019; Pelage et al., 2022; Pianka, 1974), and we indeed observed smaller isotopic niche overlap between fish and invertebrate guilds around islands with rats. On the other hand, in nutrient‐rich environments near seabird islands, niche expansion is asymmetrical and driven solely by invertebrates. Dominant competitors can alter subordinate competitors' niche width by driving them to exploit alternative, lower quality resources (Abbey‐Lee et al., 2013; Belant et al., 2010), and the nearly 52% difference in invertebrate niche width near seabird islands therefore suggests that small, motile invertebrates may be subordinate to dominant cryptobenthic fishes in this system. This is further supported by the fact that, in nutrient‐poor conditions, cryptobenthic fishes retain access to high δ^15^N resources.

Cryptobenthic fishes' life history traits make them a prime candidate for rapid exploitation of a localized resource and explain why they might exert competitive dominance over invertebrates in these systems: Rapid growth, sustained reproduction, abundant larvae, and heavily localized larval recruitment ensure that they can quickly expand their population when resources become abundant. By contrast, marine invertebrates encompass a much wider range of life history traits, with highly variable larval dispersal distances (Huber, 1985; Levin, 2006; Scheltema, 1986) and typically nonlinear growth rates. Small marine invertebrates have been associated with lower mass‐scaled metabolic rates (Banse, 1982), contrary to cryptobenthic fishes' high metabolic rates which present a competitive advantage in nutrient‐rich environments (Clarke, 1992). Near seabird islands, the high metabolic and nutrient demands of cryptobenthic fishes can be matched through uptake of seabird‐enriched resources, allowing them to maintain high growth and reproduction, to possibly outcompete or potentially prey on cryptic invertebrates for space or resources.

Several of the cryptobenthic fishes we captured are also invertivorous: Gobies, triplefins, clingfishes, cardinalfishes, and dottybacks are all known to feed on small or micro‐invertebrates (Brandl et al., 2018), which are in fact the dominant prey in *C. chagosensis*' diet. Cryptic invertebrates may therefore respond positively to nutrient enrichment but be rapidly consumed within shared microhabitats, functioning as an efficient conduit through which seabird‐derived nutrients are transferred to cryptobenthic fishes. The fact that coral‐associated trapeziid and porcellanid crab biomass declined near seabird islands—in spite of corals being well‐documented recipients of seabird nutrients in this system (Benkwitt et al., 2023; Savage, 2019)—further suggests that additional pressures are exerted onto invertebrate communities. Whether cryptobenthic fishes or other invertivorous organisms actively select nutrient‐enriched prey remains to be determined, but it is noteworthy that the invertebrates with the strongest δ^15^N signature were also characterized by the strongest decline in biomass near seabird islands. Here, we therefore suspect that any bottom‐up benefit that cryptic invertebrates might have gained from guano was outweighed by increased predation and competition pressures.

### Trophic channeling toward larger consumers

Our results have important implications for how seabird‐derived nutrients ultimately propagate to higher trophic levels on coral reefs. Here, the dottybacks *C. chagosensis*, small reef mesopredators that typically feed on invertebrates and small fishes (Ashworth et al., 2014; Palacios‐Narváez et al., 2024), switched from invertebrate prey to fish prey, exhibiting a twofold increase in cryptobenthic fish consumption near seabird islands. Because cryptobenthic fishes constitute a more protein‐dense, readily available prey resource than cryptic invertebrates, increases in their availability are likely to disproportionately benefit larger mesopredators, and this shift was indeed most pronounced for larger *C. chagosensis*. Seabird‐driven increases in fish prey availability may enhance the profitability of piscivory for larger bodied consumers through higher encounter rates, reduced foraging effort, and a more reliable prey supply associated with elevated productivity, thereby making fish a more energetically optimal prey under nutrient‐enriched conditions. *C. chagosensis* remained predominantly invertivorous, yet its increased fish consumption under seabird enrichment suggests that enhanced cryptobenthic fish biomass may partially reroute energy flow through piscivorous pathways even in mixed carnivores.

By altering the balance of cryptofaunal communities in favor of fishes over invertebrates, nutrient enrichment directs energy flow into the pathways that lead to larger piscivorous predators. Our observations are consistent with previous studies recording higher biomass of piscivores, with the second largest effect size following herbivores, and a larger effect size than invertivores near seabird islands in the Chagos Archipelago (Benkwitt et al., 2019; Graham et al., 2018). Similar to invertebrates, invertivorous fish represent a broad feeding group with a wide range of diets and may therefore present more variable responses to prey availability (Parravicini et al., 2020); invertebrate predation may also be dominated by invertivorous cryptobenthic fishes, whose spatial proximity offers more rapid access to prey than larger invertivorous fishes.

Here, 10‐fold biomass gains in piscivorous fishes far exceeded cryptobenthic fishes' twofold increase. It is worth noting that cryptobenthic fishes' contribution to reef trophodynamics far outweighs what their standing biomass might suggest due to exceptionally fast biomass turnover rates. Even modest increases in their biomass can therefore trigger disproportionate gains in higher trophic levels (Brandl et al., 2019). Some of the increase in piscivorous biomass is also likely attributable to predation on visually conspicuous fishes, and underwater visual censuses only capture some predators that specialize on cryptobenthic fishes (average predator size = 3.65 cm; average piscivorous fish size here = 35.45 cm; Mihalitsis et al., 2022). Our evidence is ultimately indirect, and targeted experiments, gut content, and isotope analysis of piscivores are likely needed to quantify the importance of cryptobenthic fishes within predatory fish diets.

Ecologically, our findings highlight how cross‐ecosystem nutrient vectors can restructure, rather than simply enhance, coral reef food webs by altering the balance between fish‐ and invertebrate‐mediated energy pathways. Seabird subsidies appear to shift resource routing and trophic interactions, and our study underscores the central role of cryptobenthic organisms in mediating these linkages. Conserving seabird populations and nesting habitats may therefore influence not only the magnitude of reef productivity but also the composition and trophic structure of adjacent marine communities, with cascading implications for food‐web dynamics.

## AUTHOR CONTRIBUTIONS

Nicholas A. J. Graham, Simon J. Brandl, Cassandra E. Benkwitt, Ruth E. Dunn, and Laura‐Li Jeannot conceptualized the study. Laura‐Li Jeannot, Ruth E. Dunn, and Cassandra E. Benkwitt collected data. M. Joyce P. Velos performed stable isotope laboratory work. Laura‐Li Jeannot performed modeling work, analyzed output data, and wrote the first draft of the manuscript. All authors contributed substantially to revisions.

## CONFLICT OF INTEREST STATEMENT

The authors declare no conflicts of interest.

## Supporting information


Appendix S1.

Appendix S2.

## Data Availability

Data and code (Jeannot, 2025) are available in Zenodo at https://doi.org/10.5281/zenodo.17186883.

## References

[ecy70453-bib-0001] Abbey‐Lee, R. N. , E. E. Gaiser , and J. C. Trexler . 2013. “Relative Roles of Dispersal Dynamics and Competition in Determining the Isotopic Niche Breadth of a Wetland Fish.” Freshwater Biology 58: 780–792.

[ecy70453-bib-0002] Ackerman, J. L. , and D. R. Bellwood . 2002. “Comparative Efficiency of Clove Oil and Rotenone for Sampling Tropical Reef Fish Assemblages.” Journal of Fish Biology 60: 893–901.

[ecy70453-bib-0003] Andrades, R. , J. M. Andrade , P. S. Jesus‐Junior , R. M. Macieira , A. F. Bernardino , T. Giarrizzo , and J.‐C. Joyeux . 2019. “Multiple Niche‐Based Analyses Reveal the Dual Life of an Intertidal Reef Predator.” Marine Ecology Progress Series 624: 131–141.

[ecy70453-bib-0004] Andrades, R. , G. C. Cardozo‐Ferreira , L. J. Benevides , C. R. Pimentel , P. L. Mancini , C. E. L. Ferreira , T. Giarrizzo , J.‐C. Joyeux , and R. M. Macieira . 2024. “Seabird Guano Reshapes Intertidal Reef Food Web in An Isolated Oceanic Islet.” Coral Reefs 43: 347–355.

[ecy70453-bib-0005] Appoo, J. , N. Bunbury , S. Jaquemet , and N. Graham . 2023. “Seabird Nutrient Subsidies Enrich Mangrove Ecosystems and are Exported to Nearby Coastal Habitats.” 10.1016/j.isci.2024.109404 PMC10952037 38510135

[ecy70453-bib-0006] Appoo, J. , N. A. J. Graham , C. W. Jones , S. Jaquemet , and N. Bunbury . 2024. “Seabird Nutrient Subsidy Alters Size and Resource Use of Functionally Important Mangrove Macroinvertebrates.” Ecosphere 15: e70121.

[ecy70453-bib-0007] Ashworth, E. C. , M. Depczynski , T. H. Holmes , and S. K. Wilson . 2014. “Quantitative Diet Analysis of Four Mesopredators from a Coral Reef.” Journal of Fish Biology 84: 1031–1045. 24641257 10.1111/jfb.12343

[ecy70453-bib-0008] Banse, K. 1982. “Mass‐Scaled Rates of Respiration and Intrinsic Growth in Very Small Invertebrates.” Marine Ecology Progress Series 9: 281–297.

[ecy70453-bib-0009] Belant, J. L. , B. Griffith , Y. Zhang , E. H. Follmann , and L. G. Adams . 2010. “Population‐Level Resource Selection by Sympatric Brown and American Black Bears in Alaska.” Polar Biology 33: 31–40.

[ecy70453-bib-0010] Benkwitt, C. , S. Wilson , and N. Graham . 2020. “Biodiversity Increases Ecosystem Functions despite Multiple Stressors on Coral Reefs.” Nature Ecology & Evolution 4: 1–8. 32424279 10.1038/s41559-020-1203-9

[ecy70453-bib-0011] Benkwitt, C. E. , K. S. I. Bistolas , J. L. DeVore , S. Ducatez , J. P. Gómez , R. Wright , M. Zubia , et al. 2025. “Re‐Connecting Ecosystems: Integrating Coral Reefs Into Monitoring of Island Restoration.” Ecological Indicators 170: 113042.

[ecy70453-bib-0012] Benkwitt, C. E. , P. Carr , S. K. Wilson , and N. A. J. Graham . 2022. “Seabird Diversity and Biomass Enhance Cross‐Ecosystem Nutrient Subsidies.” Proceedings of the Royal Society B: Biological Sciences 289: 20220195. 10.1098/rspb.2022.0195 PMC9091852 35538790

[ecy70453-bib-0013] Benkwitt, C. E. , C. D'Angelo , R. E. Dunn , R. L. Gunn , S. Healing , M. L. Mardones , J. Wiedenmann , S. K. Wilson , and N. A. J. Graham . 2023. “Seabirds Boost Coral Reef Resilience.” Science Advances 9: eadj0390. 38055814 10.1126/sciadv.adj0390 PMC10699780

[ecy70453-bib-0014] Benkwitt, C. E. , R. L. Gunn , M. Le Corre , P. Carr , and N. A. J. Graham . 2021. “Rat Eradication Restores Nutrient Subsidies from Seabirds Across Terrestrial and Marine Ecosystems.” Current Biology 31: 2704–2711.e4. 33887185 10.1016/j.cub.2021.03.104

[ecy70453-bib-0015] Benkwitt, C. E. , B. M. Taylor , M. G. Meekan , and N. A. J. Graham . 2021. “Natural Nutrient Subsidies Alter Demographic Rates in a Functionally Important Coral‐Reef Fish.” Scientific Reports 11: 12575. 34131172 10.1038/s41598-021-91884-y PMC8206227

[ecy70453-bib-0016] Benkwitt, C. E. , S. K. Wilson , and N. A. J. Graham . 2019. “Seabird Nutrient Subsidies Alter Patterns of Algal Abundance and Fish Biomass on Coral Reefs Following a Bleaching Event.” Global Change Biology 25: 2619–2632. 31157944 10.1111/gcb.14643

[ecy70453-bib-0017] Benkwitt, C. E. , A. Zora , A. Ebrahim , R. Govinden , I. D. Lange , S. Evans , M. Schulze , E. Cotton , L. Bennett , and N. A. J. Graham . 2025. “Nutrient Connectivity Via Seabirds Enhances Dynamic Measures of Coral Reef Ecosystem Function.” PLoS Biology 23: e3003222. 40627590 10.1371/journal.pbio.3003222 PMC12237027

[ecy70453-bib-0018] Brandl, S. J. , C. H. R. Goatley , D. R. Bellwood , and L. Tornabene . 2018. “The Hidden Half: Ecology and Evolution of Cryptobenthic Fishes on Coral Reefs.” Biological Reviews 93: 1846–1873. 29736999 10.1111/brv.12423

[ecy70453-bib-0019] Brandl, S. J. , L. Tornabene , C. H. R. Goatley , J. M. Casey , R. A. Morais , I. M. Côté , C. C. Baldwin , V. Parravicini , N. M. D. Schiettekatte , and D. R. Bellwood . 2019. “Demographic Dynamics of the Smallest Marine Vertebrates Fuel Coral Reef Ecosystem Functioning.” Science 364: 1189–1192. 31123105 10.1126/science.aav3384

[ecy70453-bib-0020] Brandl, S. J. , H. F. Yan , J. M. Casey , N. M. D. Schiettekatte , J. J. Renzi , A. Mercière , F. Morat , I. M. Côté , and V. Parravicini . 2025. “A Seascape Dichotomy in the Role of Small Consumers for Coral Reef Energy Fluxes.” Ecology 106: e70065. 40125610 10.1002/ecy.70065

[ecy70453-bib-0021] Carr, P. , A. M. Trevail , H. J. Koldewey , R. B. Sherley , T. Wilkinson , H. Wood , and S. C. Votier . 2023. “Marine Important Bird and Biodiversity Areas in the Chagos Archipelago.” Bird Conservation International 33: e29.

[ecy70453-bib-0022] Clarke, R. D. 1992. “Effects of Microhabitat and Metabolic Rate on Food Intake, Growth and Fecundity of Two Competing Coral Reef Fishes.” Coral Reefs 11: 199–205.

[ecy70453-bib-0023] Dunn, R. E. , N. A. J. Graham , L.‐L. Jeannot , R. Karkarey , F. J. Gonzalez‐Barrios , I. D. Lange , J. R. Fillol , R. Roche , M. Stuhr , and C. E. Benkwitt . 2025. “Active and Passive Pathways of Nutrient Transfer in Coral Reef Ecosystems.” Coral Reefs 44: 1157–1170.

[ecy70453-bib-0024] Froese, R. , and D. Pauly . 2000. Fishbase 2000: Concepts, Design and Data Sources. Los Banos, Laguna: Iclarm.

[ecy70453-bib-0025] Froese, R. , J. T. Thorson , and R. B. Reyes, Jr. 2014. “A Bayesian Approach for Estimating Length‐Weight Relationships in Fishes.” Journal of Applied Ichthyology 30: 78–85.

[ecy70453-bib-0026] Glynn, P. W. , and I. C. Enochs . 2011. “Invertebrates and Their Roles in Coral Reef Ecosystems.” In Coral Reefs: An Ecosystem in Transition, edited by Z. Dubinsky and N. Stambler , 273–325. Netherlands, Dordrecht: Springer.

[ecy70453-bib-0027] Goberdhan, L. S. , C. M. Robertson , J. P. Egerton , M. D. Fox , M. D. Johnson , N. A. J. Graham , and G. J. Williams . 2024. “Variation in Coral Rubble Cryptofauna is Scale‐Dependent and Driven by Small‐Scale Habitat Characteristics.” Marine Ecology Progress Series 750: 19–36.

[ecy70453-bib-0028] Gove, J. M. , M. A. McManus , A. B. Neuheimer , J. J. Polovina , J. C. Drazen , C. R. Smith , M. A. Merrifield , et al. 2016. “Near‐Island Biological Hotspots in Barren Ocean Basins.” Nature Communications 7: 10581. 10.1038/ncomms10581 PMC4757766 26881874

[ecy70453-bib-0029] Graham, N. A. J. , S. K. Wilson , P. Carr , A. S. Hoey , S. Jennings , and M. A. MacNeil . 2018. “Seabirds Enhance Coral Reef Productivity and Functioning in the Absence of Invasive Rats.” Nature 559: 250–253. 29995864 10.1038/s41586-018-0202-3

[ecy70453-bib-0030] Healing, S. , C. E. Benkwitt , R. E. Dunn , and N. A. J. Graham . 2024. “Seabird‐Vectored Pelagic Nutrients Integrated into Temperate Intertidal Rocky Shores.” Frontiers in Marine Science 11: 1343966.

[ecy70453-bib-0031] Herler, J. 2007. “Microhabitats and Ecomorphology of Coral‐ and Coral Rock‐Associated Gobiid Fish (Teleostei: Gobiidae) in the Northern Red Sea.” Marine Ecology 28: 82–94.

[ecy70453-bib-0032] Huber, M. E. 1985. “Population Genetics of Eight Species of Trapezia (Brachyura: Xanthidae), Symbionts of Corals.” Marine Biology 85: 23–36.

[ecy70453-bib-0033] Jackson, A. L. , R. Inger , A. C. Parnell , and S. Bearhop . 2011. “Comparing Isotopic Niche Widths Among and Within Communities: SIBER—Stable Isotope Bayesian Ellipses in R.” Journal of Animal Ecology 80: 595–602. 21401589 10.1111/j.1365-2656.2011.01806.x

[ecy70453-bib-0034] Jackson, D. A. , and H. H. Harvey . 1993. “Fish and Benthic Invertebrates: Community Concordance and Community–Environment Relationships.” Canadian Journal of Fisheries and Aquatic Sciences 50: 2641–2651.

[ecy70453-bib-0035] Jeannot, L.‐L. 2025. “lljeannot/seabirds_cryptofauna: Data and Code v1 (v1.0.0).” Zenodo. 10.5281/zenodo.17186883

[ecy70453-bib-0036] Jeannot, L.‐L. , J. P. Lozano‐Peña , A. Zora , S. J. Brandl , and N. A. J. Graham . 2025. “Seabird‐Derived Nutrients Influence Feeding Pathways and Body Size in Cryptobenthic Reef Fishes.” Proceedings of the Royal Society B: Biological Sciences 292: 20250539. 10.1098/rspb.2025.0539 PMC12308344 40628474

[ecy70453-bib-0037] Jones, H. P. , J. Appoo , C. E. Benkwitt , S. B. Borrelle , R. E. Dunn , H. E. Epstein , L. A. Fowlke , et al. 2025. “The Circular Seabird Economy is Critical for Oceans, Islands and People.” Nature Reviews Biodiversity 1: 689–702.

[ecy70453-bib-0038] Jones, H. P. , B. R. Tershy , E. S. Zavaleta , D. A. Croll , B. S. Keitt , M. E. Finkelstein , and G. R. Howald . 2008. “Severity of the Effects of Invasive Rats on Seabirds: A Global Review.” Conservation Biology: The Journal of the Society for Conservation Biology 22: 16–26. 18254849 10.1111/j.1523-1739.2007.00859.x

[ecy70453-bib-0039] Kolb, G. S. , J. Ekholm , and P. A. Hambäck . 2010. “Effects of Seabird Nesting Colonies on Algae and Aquatic Invertebrates in Coastal Waters.” Marine Ecology Progress Series 417: 287–300.

[ecy70453-bib-0040] Levin, L. A. 2006. “Recent Progress in Understanding Larval Dispersal: New Directions and Digressions.” Integrative and Comparative Biology 46: 282–297. 21672742 10.1093/icb/icj024

[ecy70453-bib-0041] Lin, Y.‐J. , L. Rabaoui , A. U. Basali , M. Lopez , R. Lindo , P. K. Krishnakumar , M. A. Qurban , et al. 2021. “Long‐Term Ecological Changes in Fishes and Macro‐Invertebrates in the World's Warmest Coral Reefs.” Science of the Total Environment 750: 142254. 33182216 10.1016/j.scitotenv.2020.142254

[ecy70453-bib-0042] McCauley, D. J. , P. A. DeSalles , H. S. Young , R. B. Dunbar , R. Dirzo , M. M. Mills , and F. Micheli . 2012. “From Wing to Wing: The Persistence of Long Ecological Interaction Chains in Less‐Disturbed Ecosystems.” Scientific Reports 2: 409. 22624091 10.1038/srep00409 PMC3354671

[ecy70453-bib-0043] Mihalitsis, M. , R. A. Morais , and D. R. Bellwood . 2022. “Small Predators Dominate Fish Predation in Coral Reef Communities.” PLoS Biology 20: e3001898. 36445867 10.1371/journal.pbio.3001898 PMC9707750

[ecy70453-bib-0044] Morais, R. A. , and D. R. Bellwood . 2020. “Principles for Estimating Fish Productivity on Coral Reefs.” Coral Reefs 39: 1221–1231.

[ecy70453-bib-0045] Oksanen, J. , G. L. Simpson , F. G. Blanchet , R. Kindt , P. Legendre , P. R. Minchin , R. B. O'Hara , et al. 2001. “vegan: Community Ecology Package.”

[ecy70453-bib-0046] Otero, X. L. , S. De La Peña‐Lastra , A. Pérez‐Alberti , T. O. Ferreira , and M. A. Huerta‐Diaz . 2018. “Seabird Colonies as Important Global Drivers in the Nitrogen and Phosphorus Cycles.” Nature Communications 9: 246. 10.1038/s41467-017-02446-8 PMC5780392 29362437

[ecy70453-bib-0047] Palacios‐Narváez, S. , D. J. Coker , E. Aylagas , M. S. Justo , V. Nunes‐Peinemann , M. D. Tietbohl , C. Bocanegra , C. P. Antony , and M. L. Berumen . 2024. “Dietary Partitioning among Three Cryptobenthic Reef Fish Mesopredators Revealed by Visual Analysis, Metabarcoding of Gut Content, and Stable Isotope Analysis.” Environmental DNA 6: e541.

[ecy70453-bib-0048] Parravicini, V. , J. M. Casey , N. M. D. Schiettekatte , S. J. Brandl , C. Pozas‐Schacre , J. Carlot , G. J. Edgar , et al. 2020. “Delineating Reef Fish Trophic Guilds with Global Gut Content Data Synthesis and Phylogeny.” PLoS Biology 18: e3000702. 33370276 10.1371/journal.pbio.3000702 PMC7793298

[ecy70453-bib-0049] Pearson, D. E. , T. J. Clark , and P. G. Hahn . 2022. “Evaluating Unintended Consequences of Intentional Species Introductions and Eradications for Improved Conservation Management.” Conservation Biology 36: e13734. 33734489 10.1111/cobi.13734 PMC9291768

[ecy70453-bib-0050] Pelage, L. , F. Lucena‐Frédou , L. N. Eduardo , F. Le Loc'h , A. Bertrand , A. S. Lira , and T. Frédou . 2022. “Competing with Each Other: Fish Isotopic Niche in Two Resource Availability Contexts.” Frontiers in Marine Science 9: 975091.

[ecy70453-bib-0051] Pianka, E. R. 1974. “Niche Overlap and Diffuse Competition.” Proceedings of the National Academy of Sciences 71: 2141–2145. 10.1073/pnas.71.5.2141 PMC388403 4525324

[ecy70453-bib-0052] Plaisance, L. , K. Matterson , K. Fabricius , S. Drovetski , C. Meyer , and N. Knowlton . 2021. “Effects of Low pH on the Coral Reef Cryptic Invertebrate Communities near CO_2_ Vents in Papua New Guinea.” PLoS One 16: e0258725. 34910721 10.1371/journal.pone.0258725 PMC8673656

[ecy70453-bib-0053] Post, D. M. 2002. “Using Stable Isotopes to Estimate Trophic Position: Models, Methods, and Assumptions.” Ecology 83: 703–718.

[ecy70453-bib-0054] Rayner, R. F. , and E. A. Drew . 1984. “Nutrient Concentrations and Primary Productivity at the Peros Banhos and Salomon Atolls in the Chagos Archipelago.” Estuarine, Coastal and Shelf Science 18: 121–132.

[ecy70453-bib-0055] Sandin, S. A. , and I. Williams . 2010. “Trophic Classifications of Reef Fishes from the Tropical U.S. Pacific (Version 1.0).”

[ecy70453-bib-0056] Savage, C. 2019. “Seabird Nutrients are Assimilated by Corals and Enhance Coral Growth Rates.” Scientific Reports 9: 4284. 30862902 10.1038/s41598-019-41030-6 PMC6414626

[ecy70453-bib-0057] Scheltema, R. S. 1986. “Long‐Distance Dispersal by Planktonic Larvae of Shoal‐Water Benthic Invertebrates Among Central Pacific Islands.” Bulletin of Marine Science 39: 171–186.

[ecy70453-bib-0058] Stella, J. , M. Pratchett , P. Hutchings , and G. Jones . 2011. “Coral‐Associated Invertebrates: Diversity, Ecological Importance and Vulnerability to Disturbance.” Oceanography and Marine Biology 49: 43–104.

[ecy70453-bib-0059] Stier, A. C. , and M. Leray . 2014. “Predators Alter Community Organization of Coral Reef Cryptofauna and Reduce Abundance of Coral Mutualists.” Coral Reefs 33: 181–191.

[ecy70453-bib-0060] Stock, B. C. , A. L. Jackson , E. J. Ward , A. C. Parnell , D. L. Phillips , and B. X. Semmens . 2018. “Analyzing Mixing Systems Using a New Generation of Bayesian Tracer Mixing Models.” PeerJ 6: e5096. 29942712 10.7717/peerj.5096 PMC6015753

[ecy70453-bib-0061] Stöhr, S. , T. D. O'Hara , and B. Thuy . 2012. “Global Diversity of Brittle Stars (Echinodermata: Ophiuroidea).” PLoS One 7: e31940. 22396744 10.1371/journal.pone.0031940 PMC3292557

[ecy70453-bib-0062] Stuart, C. E. , S. J. Pittman , K. A. Stamoulis , C. E. Benkwitt , H. E. Epstein , N. A. J. Graham , A. C. Smith , et al. 2025. “Seascape Configuration Determines Spatial Patterns of Seabird‐Vectored Nutrient Enrichment to Coral Reefs.” Ecography 2025: e07863.

[ecy70453-bib-0063] Takada, Y. , O. Abe , and T. Shibuno . 2007. “Colonization Patterns of Mobile Cryptic Animals into Interstices of Coral Rubble.” Marine Ecology Progress Series 343: 35–44.

[ecy70453-bib-0064] Werding, B. , B. Christensen , and A. Hiller . 2016. “Three Way Symbiosis Between a Goby, a Shrimp, and a Crab.” Marine Biodiversity 46: 897–900.

[ecy70453-bib-0065] Wyżga, B. , A. Amirowicz , P. Oglęcki , H. Hajdukiewicz , A. Radecki‐Pawlik , J. Zawiejska , and P. Mikuś . 2014. “Response of Fish and Benthic Invertebrate Communities to Constrained Channel Conditions in a Mountain River: Case Study of the Biała, Polish Carpathians.” Limnologica 46: 58–69.

