# Supplementary material for: Contrasting cryptofaunal responses to seabird nutrient inputs illuminate coral reef productivity pathways
Source: Ecology. 2026 Jul 22;107(7):e70453. doi: 10.1002/ecy.70453 (PMC13390649; doi:10.1002/ecy.70453)
Supplement: Supplementary file 1 — Appendix S1. [file ECY-107-e70453-s002.pdf]

Appendix S1

Contrasting cryptofaunal responses to seabird nutrient inputs illuminate coral reef productivity pathways

Laura-Li Jeannot, Ruth E. Dunn, Joyce Velos, Gareth J. Williams, Cassandra E. Benkwitt, Nicholas A. J. Graham, Simon J. Brandl

Ecology

Section S1. Field procedure for cryptofauna collection.

A mesh net (hole size: ~ 1 mm) and a 8.5 m^2 circular tarpaulin sheet, both weighed with a metal chain wrapped around their base, were laid over the selected coral outcrop by teams consisting of 3 trained divers, one of which was consistently present for all dives. The coral outcrop under the tarpaulin sheet was then sprayed with a solution of 1:5 clove oil (Essential Oils Direct, UK) to 95% ethanol. Clove oil is well established as an anaesthetic for both fishes (Hoskonen and Pirhonen 2004) and invertebrates (Stier and Leray 2014). After ~ 5 minutes, the diving team collected samples underneath the net, placing them in clear slider storage bags. The team circled around the outcrop, gradually peeling the tarpaulin sheet and net back whilst collecting samples, until the sheet and net were removed and no samples were found for another ~ 5 minutes. All samples were then brought back to the surface and frozen until processing.

Section S2. Stable isotope analyses.

All individuals were photographed, weighed, measured, and identified to the highest taxonomic level possible. This was at species level for fish and family level (90.3% of all sampled invertebrates) and above (9.7%) for invertebrates, due to comparatively limited taxonomic resolution for small invertebrates in the region. Average size and weight per taxa and invertebrate length metrics are presented in Table S2. Whole-body samples were used for

invertebrates, including shells, due to limited available material. All tissue types (bones, scales, muscles) were used for fish as well. Samples were ground to a fine powder using a bead mill. For fish and invertebrate samples, ~0.5 and ~1.35 mg, respectively, were weighed and sealed into tin capsules. Isotopic composition was measured via combustion in an automated system for nitrogen- and carbon-isotope measurements using a Thermo Fisher Scientific Flash EA-Isolink CNSOH elemental analyzer connected to a Thermo Fisher Scientific Delta V Plus isotope-ratio-mass-spectrometer. Analytical precision was calculated as the mean within-run standard deviation from USGS-40 and USGS-41a and was < 0.2 ‰ for both carbon and nitrogen isotope composition.

Stable isotope values are reported using standard delta (δ) notation in parts per thousand (‰), as described in the following equation:

$$\delta X = \left(\frac{R_{sample}}{R_{standard}} - 1\right) \times 1000$$

where X is ^{13}C or ^{15}N and R is the ratio of the heavy to light isotope (i.e., $^{13}C/^{12}C$ or $^{15}N/^{14}N$) of the sample and international standards (V-PDB and AIR for carbon and nitrogen, respectively) (McKinney et al. 1950, Peterson and Fry 1987). Fish C:N ratios indicated samples had < 5% of lipid content (mean C:N ratio = 3.51 ± 0.18 SD), thus eliminating potential bias introduced by high lipid content (Post et al. 2007). Invertebrate $\delta^{13}C$ was lipid-normalized using the McConaughey & McRoy model (McConnaughey and McRoy 1979, Ouellet et al. 2024), following:

$$\delta^{13}C_{lipid-free} = \delta^{13}C_{bulk} + D \times \left[1 + \frac{3.90}{\left(1 + \frac{287}{L}\right)}\right]$$

$$L = \frac{93}{1 + (0.246 \times C{:}N_{bulk} - 0.775)^{-1}}$$

Section S3: Data analysis.

Isotopic niche models were run using four chains of 2,000 iterations, 1,000 burn-in samples, and a thinning factor of 1. We assessed MCMC convergence for each ellipse using Gelman-Rubin potential scale reduction factors ($\hat{R}$) computed in coda from the chain-specific JAGS output saved by SIBER; all parameters had $\hat{R} < 1.05$.

All Bayesian models were also run with four chains and 2,000 iterations including 1,000 burn-in samples, using brms v 2.22.0 (Bürkner 2017). Model validation was performed using visual evaluation of chain convergence and posterior predictive checks. Models were run with default priors unless specified in Table S3, in which case prior predictive checks were performed.

Mixing models were run in MixSIAR using the "very long" MCMC settings (chain length = 300,000; burn-in = 200,000, 3 chains), but with a thinning factor of 5. Convergence was assessed using Gelman–Rubin diagnostics ($\hat{R}$), and all parameters had $\hat{R} < 1.05$.

Density and biomass models were also run at cryptobenthic fish family level in addition to species level, with a small constant set to the order of magnitude of the smallest non-zero observation.

For piscivorous and invertivorous productivity models, species trait data was extracted from Froese and Pauly (2000) and Morais and Bellwood (2018), and sea surface temperature was set at 28°C which corresponds to the average sea surface temperature in the Chagos Archipelago (Sheppard et al. 2012, Benkwitt et al. 2020).

All analyses were done in R 4.5.0 (R Core Team 2025).

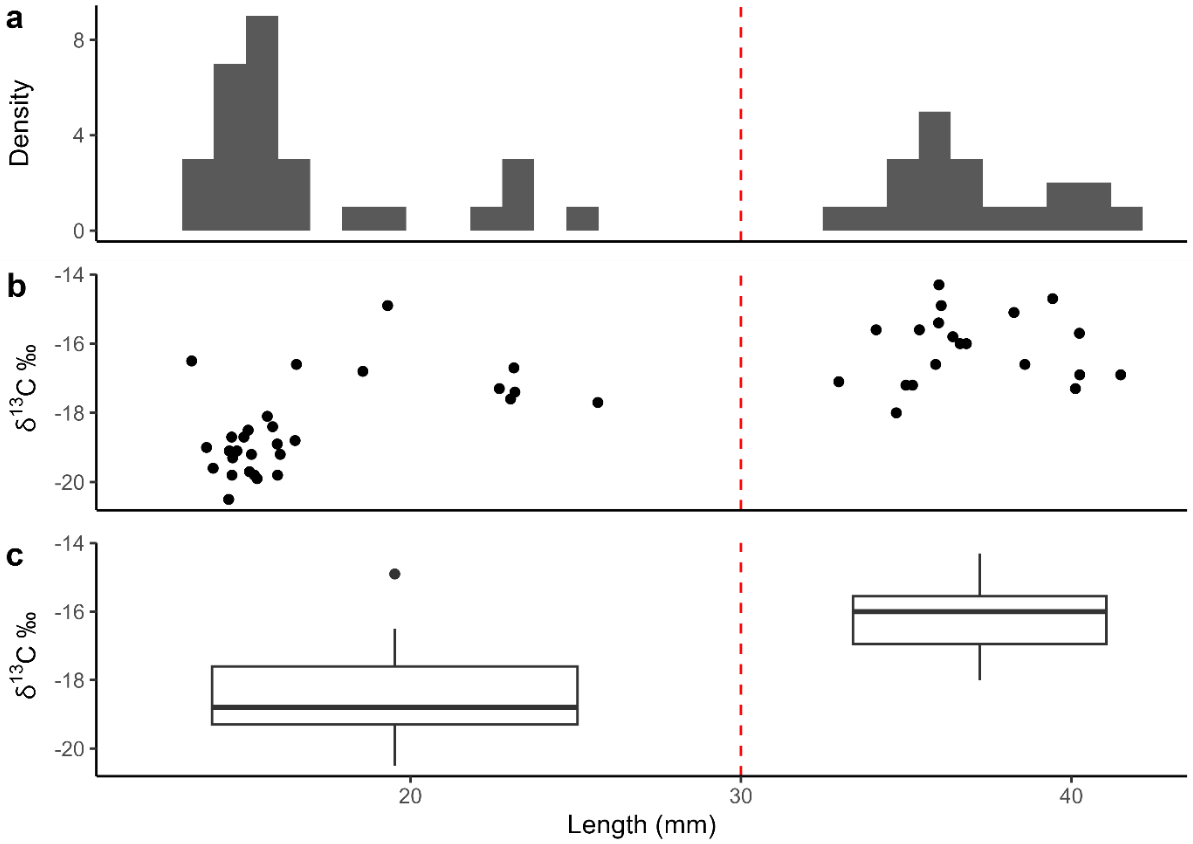

Figure S1: Relationship between total length and $\delta^{13}C$ in *Chlidichthys chagosensis*. (a) Histogram showing the frequency distribution of lengths; (b) Scatterplot of $\delta^{13}C$ values against individual length; (c) Boxplots of $\delta^{13}C$ for individuals <30 mm and >30 mm. The red dashed line indicates the 30 mm threshold used to separate size classes.

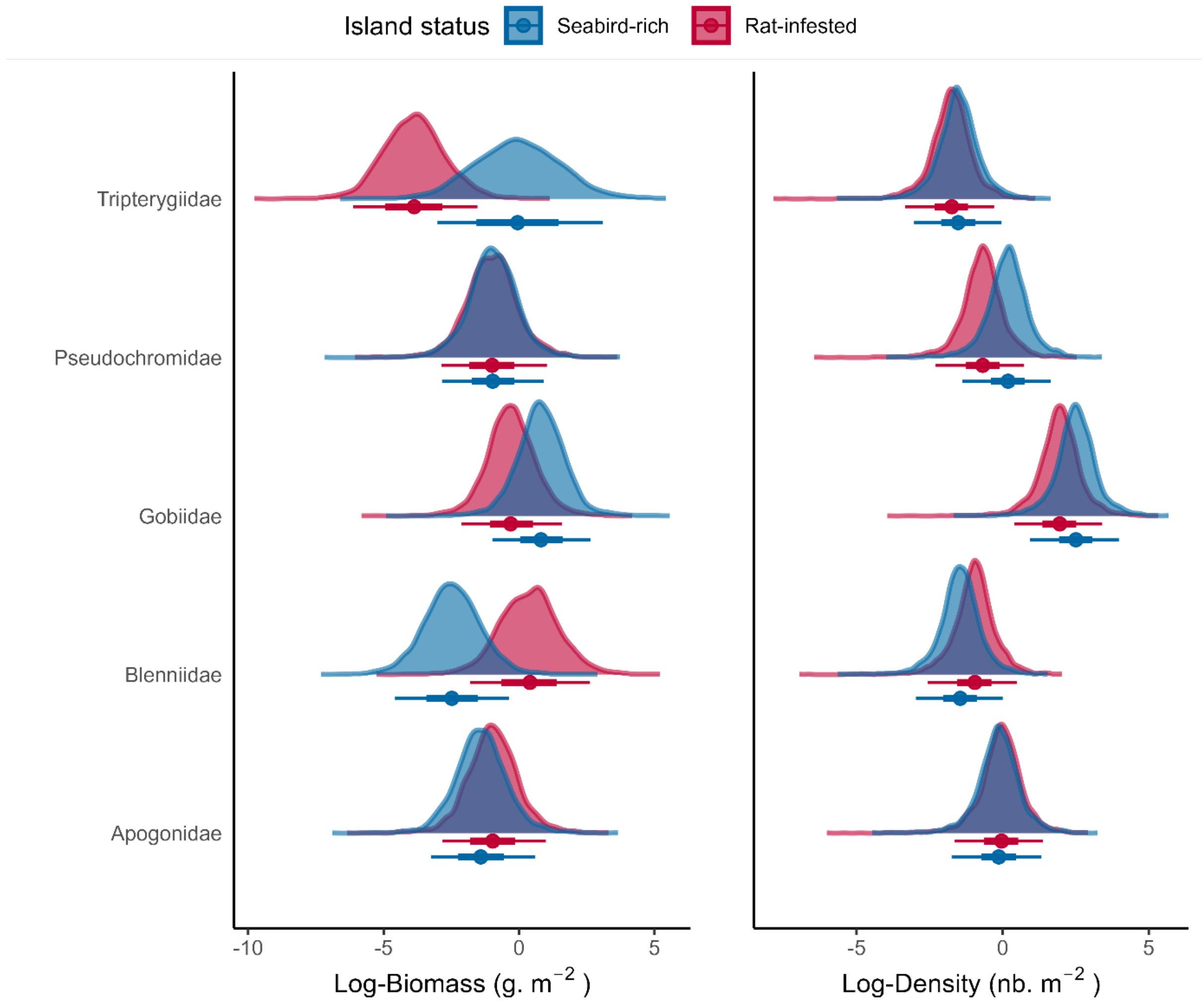

Figure S2: Density and biomass of cryptobenthic fishes by family. Density curves and caterpillar plots (50% and 95% credible intervals) represent fitted values from Bayesian linear models based on 1,000 posterior draws.

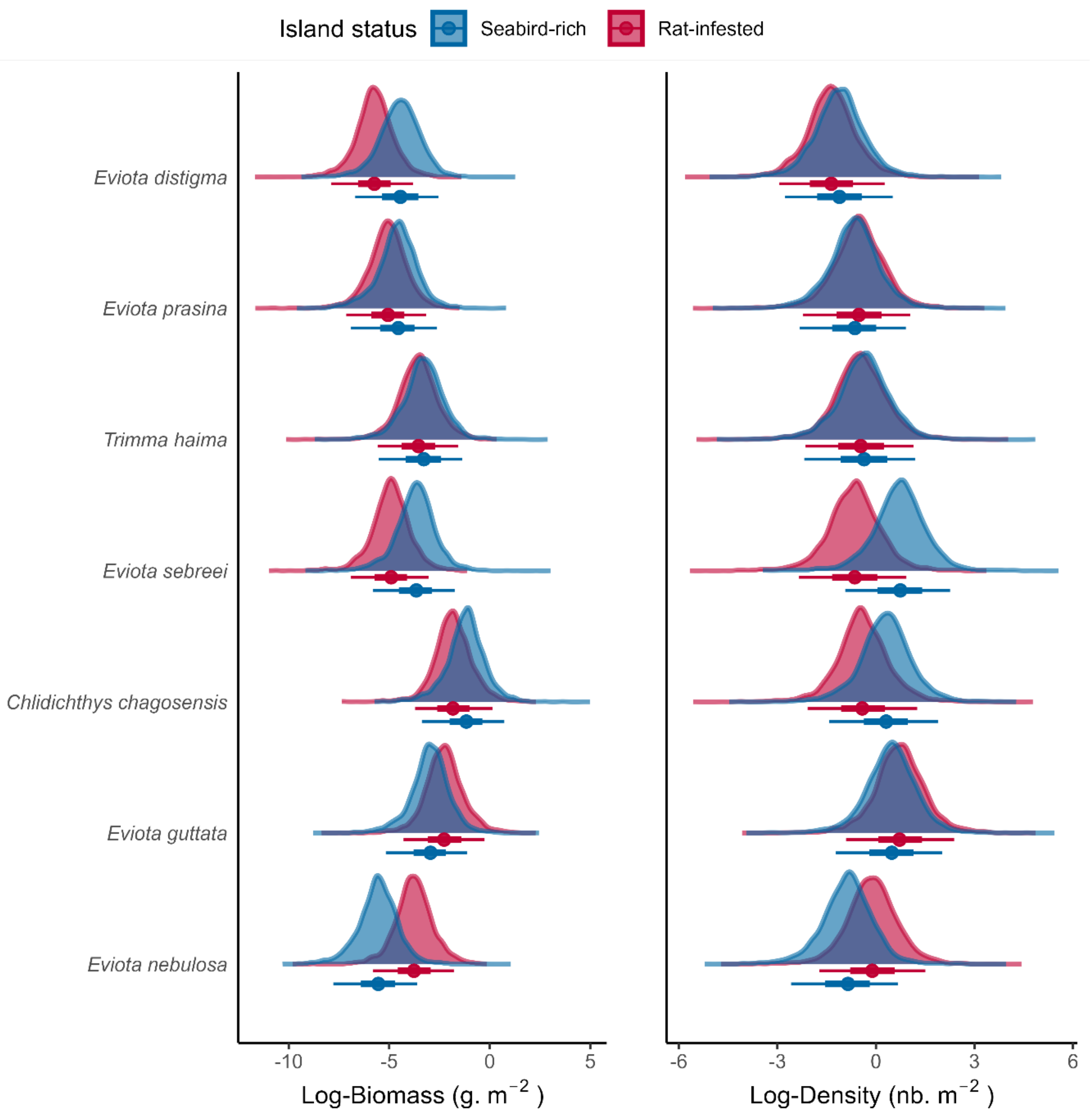

Figure S3: Density and biomass of cryptobenthic fishes by species. Only species chosen for stable isotope analysis are represented. Density curves and caterpillar plots (50% and 95% credible intervals) represent fitted values from Bayesian linear models based on 1,000 posterior draws.

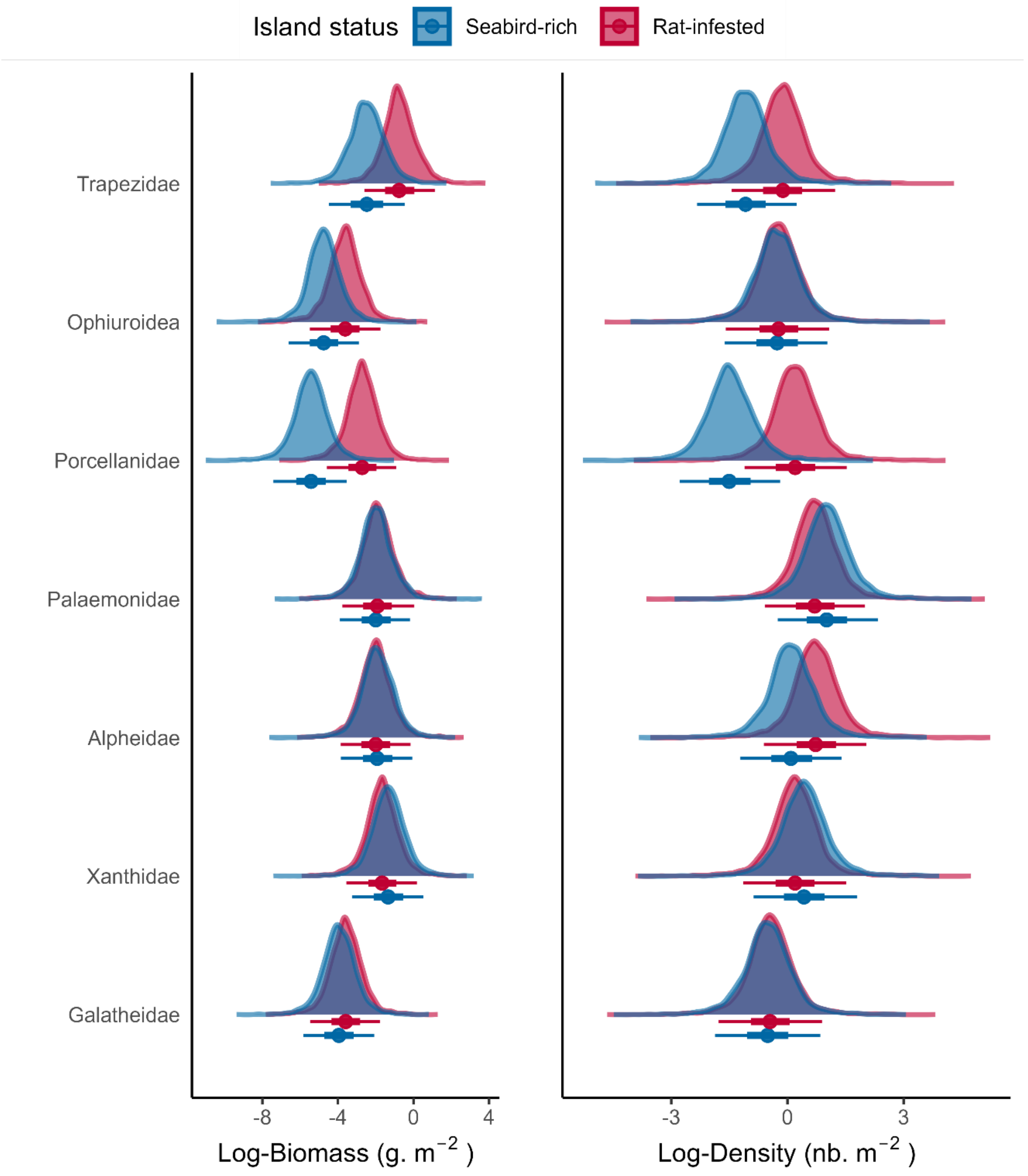

Figure S4: Density and biomass of invertebrates by family. Density curves and caterpillar plots (50% and 95% credible intervals) represent fitted values from Bayesian linear models based on 1,000 posterior draws.

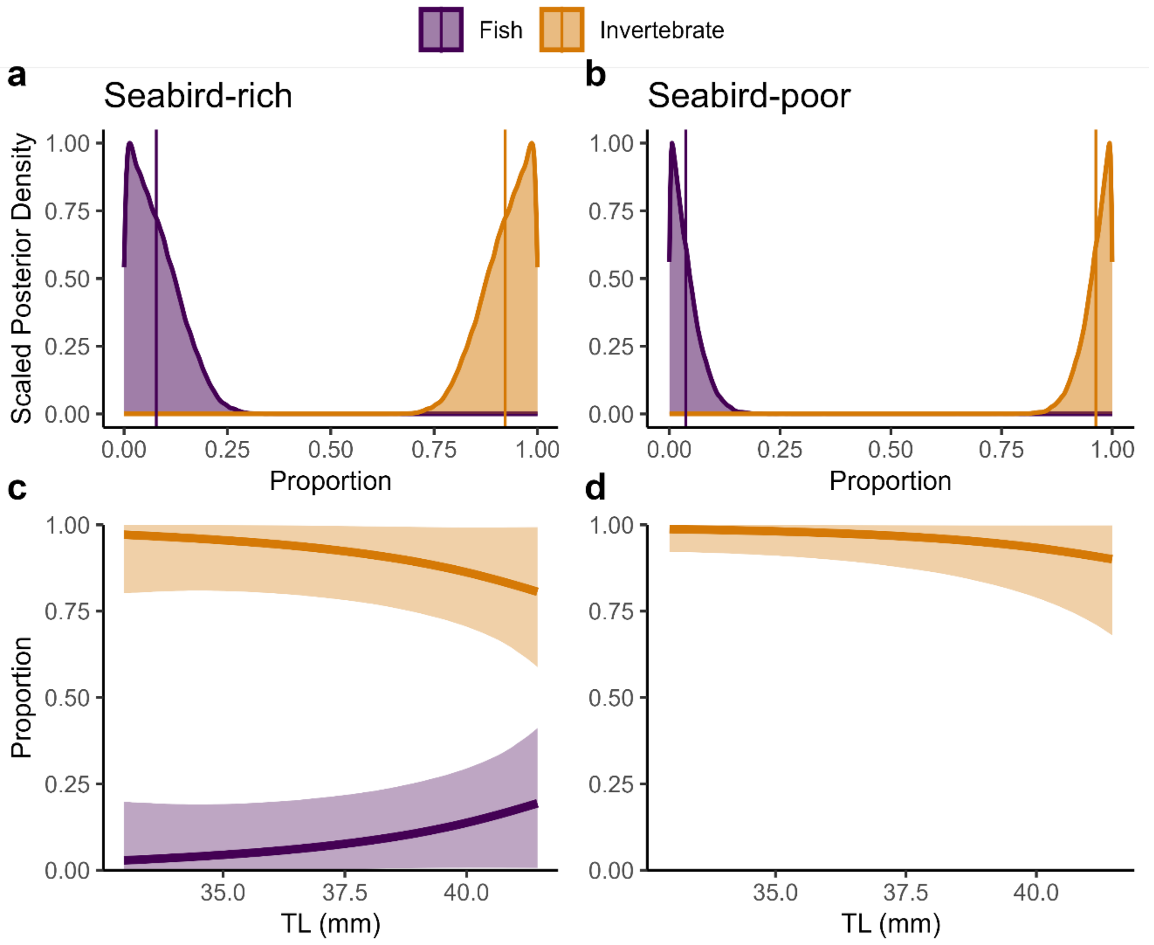

Figure S5: Posterior distributions for diet proportions for *Chlidicthys chagosensis*. (a) and (c) represent scaled posterior density for overall diet proportions, and diet proportions according to total length respectively in the seabird-rich location. (b) and (d) represent scaled posterior density for overall diet proportions, and diet proportions according to total length respectively in seabird-poor location. Lines represent posterior medians and shading represents the 90% credible intervals.

Table S1: Sampling site list. The Analyses column indicates whether samples from this sampling site were included only in stable isotope analyses (SIA), or in all analyses (All). Seabird densities are from (Carr et al. 2021) and correspond to the number of breeding pairs per island.

ID	Date	Atoll	Island	Location	Island status	Seabird density	CSL	Analyses
A	17-Oct	Salomon	Passe	Lagoon	Seabird-rich	498	250	SIA
B	18-Oct	Salomon	Ile Anglaise	Lagoon	Rat-infested	46	250	All
C	18-Oct	Salomon	Ile Anglaise	Lagoon	Rat-infested	46	210	All
D	19-Oct	Salomon	Ile Anglaise	Lagoon	Rat-infested	46	180	All
E	19-Oct	Salomon	Passe	Lagoon	Seabird-rich	498	240	All
F	20-Oct	Salomon	Passe	Lagoon	Seabird-rich	498	230	All
G	20-Oct	Salomon	Passe	Lagoon	Seabird-rich	498	250	All
H	22-Oct	Peros Banhos	Grande Ile Coquillage	Lagoon	Seabird-rich	40,525	220	SIA
I	22-Oct	Peros Banhos	Grande Ile Coquillage	Lagoon	Seabird-rich	40,525	180	All
J	23-Oct	Peros Banhos	Grande Ile Coquillage	Lagoon	Seabird-rich	40,525	220	All
K	23-Oct	Peros Banhos	Grande Ile Coquillage	Lagoon	Seabird-rich	40,525	210	All
L	24-Oct	Peros Banhos	Ile Anglaise	Lagoon	Rat-infested	40	210	All
M	25-Oct	Peros Banhos	Ile Anglaise	Lagoon	Rat-infested	40	250	SIA
N	27-Oct	Peros Banhos	Ile Yeye	Lagoon	Rat-infested	147	250	All
O	27-Oct	Peros Banhos	Ile Yeye	Lagoon	Rat-infested	147	220	All
P	28-Oct	Great Chagos Bank	Middle Brother	Lagoon	Seabird-rich	32,275	150	All
Q	28-Oct	Great Chagos Bank	Middle Brother	Lagoon	Seabird-rich	32,275	150	All
R	29-Oct	Great Chagos Bank	Middle Brother	Lagoon	Seabird-rich	32,275	180	All
S	29-Oct	Great Chagos Bank	Middle Brother	Lagoon	Seabird-rich	32,275	210	All

T	30-Oct	Great Chagos Bank	Eagle Island	Outer reef	Rat-infested	81	200	All
U	30-Oct	Great Chagos Bank	Eagle Island	Outer reef	Rat-infested	81	240	All
V	31-Oct	Great Chagos Bank	Eagle Island	Outer reef	Rat-infested	81	220	All
W	31-Oct	Great Chagos Bank	Eagle Island	Outer reef	Rat-infested	81	260	All

Table S2: Abundance of cryptofauna per island status. N represents the number of individuals sampled from sites included in stable isotope analyses.

Guild	Taxon	Seabird-rich N	Rat-infested N
Fish	*Chlidichthys chagosensis*	34	16
	Eviota distigma	7	11
	Eviota guttata	46	42
	Eviota nebulosa	15	16
	Eviota prasina	35	47
	Eviota sebreei	48	19
	Trimma haima	16	13
	Total	201	164
Invertebrate	Alpheidae	30	73
	Galatheidae	19	24
	Ophiuroideae	18	25
	Palaemonidae	67	62
	Porcellanidae	8	51
	Trapeziidae	7	30
	Xanthidae	38	35
	Total	187	300

Table S3 (next page): Prior specifications. Priors were selected on published literature. Prior predictive checks were conducted by running a model with sample_prior = “only”, visualizing prior predictions, and generating 100 draws from the prior predictive distribution to examine simulated values.

Model	Distribution family	Taxon	Parameter	Prior	Reference
$\delta^{15}N$ ~ Treatment + (1|Atoll/Island)<br>$\delta^{15}N$ ~ Treatment*Species + (1|Atoll/Island)<br>$\delta^{15}N$ ~ Treatment*Total length + (1|Atoll/Island) + (1|Species)	Gaussian	Cryptobenthic fishes	Intercept	normal(10, 2.5)	Jeannot et al. (2025)
			β	normal(-0.64, 0.5)	
			σ	exponential(1)	
$\delta^{13}C$ & $\delta^{15}N$ (SIBER)	Bivariate Normal	All cryptofauna	μ	normal(0, $(10^{-3})^{-1}$)	
			Σ	Inverse-Wishart($R = I_2$, $k = 2$)	
Productivity ~ Treatment + (1/Atoll|Year) + (1|Year)	Gamma(link = "log")	Piscivorous fishes	Intercept	normal(3.5, 0.5)	Benkwitt et al. (2020)
			β	normal(-1.60, 0.67)	
			σ	normal(0, 0.5)	
			γ	gamma(2, 0.1)	
Biomass ~ Treatment + (1/Atoll|Year) + (1|Year)	Gamma(link = "log")	Piscivorous fishes	Intercept	normal(3, 0.5)	Benkwitt et al. (2020)
			β	normal(-1.85, 0.70)	
			σ	normal(0, 0.5)	
			γ	gamma(2, 0.1)	
Productivity ~ Treatment + (1/Atoll|Year) + (1|Year)	Gamma(link = "log")	Invertivorous fishes	Intercept	normal(3, 0.5)	Benkwitt et al. (2020)
			β	normal(0.48, 0.41)	
			σ	normal(0, 0.5)	
			γ	gamma(2, 0.1)	
Biomass ~ Treatment + (1/Atoll|Year) + (1|Year)	Gamma(link = "log")	Invertivorous fishes	Intercept	normal(2, 0.5)	Benkwitt et al. (2020)
			β	normal(0.31, 0.38)	
			σ	normal(0, 0.5)	
			γ	gamma(2, 0.1)	

Table S4: Summary data for fish and invertebrate sizes. N represents the number of individuals that were sampled from sites included in all analyses. Length is in mm, weight is in g. Only invertebrate families with N > 30 are presented.

**Guild**	**Family**	**N**	**Length metric**	**Mean Length**	**SD Length**	**Minimum length**	**Weight**	**SD Weight**
Fish	Apogonidae	67	Total length	26.9	18.0	8.6	0.7	2.4
	Blenniidae	18		32.7	15.1	14.3	0.8	1.4
	Gobiidae	560		16.4	9.0	6.0	0.1	0.5
	Pseudochromidae	49		24.9	10.4	13.4	0.3	0.3
	Syngnathidae	1		40.3	NA	40.3	0.1	NA
	Tripterygiidae	8		20.6	6.2	13.1	0.1	0.1
	All	703		18.5	11.3	6.0	0.2	0.9
Invertebrate	Palaemonidae	126	Total length	14.1	4.7	5.7	0.1	0.1
	Alpheidae	98	Total length	12.9	5.5	5.8	0.1	0.2
	Xanthidae	72	Carapace width	6.1	3.1	2.0	0.2	0.2
	Porcellanidae	56	Carapace width	3.6	0.9	1.9	0.1	0.1
	Ophiuroidae	42	Arm diameter	1.7	1.2	0.4	NA	NA
	Galatheidae	40	Total length	8.5	2.4	3.5	0.0	0.0
	Trapeziidae	37	Carapace width	8.0	3.1	2.6	0.4	0.4
	All	622		10.5	7.0	0.4	0.1	0.2

Table S5: Hypotheses tests for $\delta^{15}N$ values. Estimate, error, lower CI, upper CI, evidence ratio and posterior probability refer to the hypothesis that $\delta^{15}N$ values are higher in the seabird-rich islands compared to the rat-infested islands.

**Taxa**	**Estimate**	**Est. Error**	**CI.Lower**	**CI.Upper**	**Evid.Ratio**	**Post.Prob**
Cryptobenthic fishes (overall)	0.34	0.25	-0.05	0.79	12.16	0.92
Chlidichthys chagosensis	0.33	0.29	-0.11	0.82	7.28	0.88
Eviota guttata	0.24	0.33	-0.25	0.81	3.42	0.77
Eviota distigma	0.69	0.41	0.02	1.39	21.73	0.96
Eviota nebulosa	0.08	0.39	-0.52	0.73	1.28	0.56
Eviota prasina	0.51	0.35	-0.03	1.08	15.67	0.94
Eviota sebreei	0.32	0.33	-0.19	0.89	5.63	0.85
Trimma haima	0.63	0.38	0.04	1.27	24.16	0.96
Cryptobenthic invertebrates (overall)	0.37	0.55	-0.37	1.18	4.19	0.81
Alpheidae	0.08	0.41	-0.52	0.73	1.36	0.58
Porcellanidae	0.26	0.73	-0.92	1.46	1.81	0.64
Galatheidae	-0.29	0.55	-1.17	0.6	0.4	0.28
Palaemonidae	0.26	0.4	-0.3	0.86	3.69	0.79
Trapeziidae	0.8	0.58	-0.15	1.77	12.16	0.92
Xanthidae	0.01	0.45	-0.65	0.7	1.05	0.51
Ophiuroideae	0.62	0.52	-0.2	1.45	8.98	0.9

Table S6: Cryptobenthic fish SIMPER analysis results. Only species with a p-value below 0.05 are presented. Ratio refers to the number of individuals present in the rat-infested sampling sites to the number present in the seabird-rich sampling sites.

**Species**	**Average**	**SD**	**Ratio**	**Rat-infested Average**	**Seabird-rich Average**	**p-value**
Amblygobius phalaena	0.016	0.022	0.721	0.0	1.5	0.021
Pleurosicya mossambica	0.038	0.046	0.830	0.2	3.6	0.023
Fowleria marmorata	0.004	0.008	0.477	0.0	0.3	0.028
Fusigobius neophytus	0.032	0.027	1.230	0.6	2.4	0.030
Amblygobius albimaculatus	0.004	0.006	0.323	0.0	0.2	0.031
Apogon indicus	0.002	0.015	0.575	0.0	0.1	0.039
Nectamia savayensis	0.009	0.012	0.325	0.0	0.8	0.040
Helcogramma fuscopinna	0.004	0.014	0.751	0.0	0.2	0.044
Pleurosicya labiata	0.011	0.006	0.328	0.1	0.8	0.048

Table S7: Cryptic invertebrate SIMPER analysis results. Only families with a p-value below 0.05 are presented. Ratio refers to the number of individuals present in the rat-infested sampling sites to the number present in the seabird-rich sampling sites.

**Taxa**	**Average**	**SD**	**Ratio**	**Rat-infested Average**	**Seabird-rich Average**	**p-value**
Cypraeidae	0.003	0.010	0.316	0.1	0	0.017
Porcellanidae	0.073	0.079	0.930	5.1	0.5	0.025
Trapeziidae	0.046	0.043	1.069	3.0	0.7	0.045

Table S8: SIBER analyses results. TA and SEAc correspond to Total Area and corrected Standard Ellipse Area respectively.

**Guild**	**Taxon**	**TASeabird-rich**	**TARat-infested**	**SEAcSeabird-rich**	**SEAcRat-infested**
Fish	*Chlidichthys chagosensis*	7.46	7.86	2.73	3.79
	Eviota distigma	4.02	9.19	4.20	8.17
	Eviota guttata	7.91	4.86	2.84	2.29
	Eviota nebulosa	3.23	8.20	1.55	3.80
	Eviota prasina	3.20	5.20	1.65	1.66
	Eviota sebreei	10.77	7.80	4.23	3.52
	Trimma haima	2.11	2.75	0.86	1.60
	Total	19.55	21.07	4.33	4.63
Invertebrate	Alpheidae	11.50	19.58	4.21	4.29
	Galatheidae	9.25	14.04	7.13	5.82
	Ophiuroideae	47.49	20.50	26.85	9.87
	Palaemonidae	25.35	32.98	8.57	6.13
	Porcellanidae	1.47	13.35	5.32	4.16
	Trapeziidae	23.25	22.48	20.61	8.22
	Xanthidae	30.81	42.81	8.67	14.45
	Total	97.29	81.21	16.64	10.31

References

Benkwitt, C., S. Wilson, and N. Graham. 2020. Biodiversity increases ecosystem functions despite multiple stressors on coral reefs. Nature Ecology & Evolution 4:1–8.

Brandl, S. J., L. Tornabene, C. H. R. Goatley, J. M. Casey, R. A. Morais, I. M. Côté, C. C. Baldwin, V. Parravicini, N. M. D. Schiettekatte, and D. R. Bellwood. 2019. Demographic dynamics of the smallest marine vertebrates fuel coral reef ecosystem functioning. Science 364:1189–1192.

Bürkner, P.-C. 2017. brms: An R Package for Bayesian Multilevel Models Using Stan. Journal of Statistical Software 80:1–28.

Carr, P., S. Votier, H. Koldewey, B. Godley, H. Wood, and M. a. C. Nicoll. 2021. Status and phenology of breeding seabirds and a review of Important Bird and Biodiversity Areas in the British Indian Ocean Territory. Bird Conservation International 31:14–34.

Froese, R., and D. Pauly. 2000. Fishbase 2000: Concepts, Design and Data Sources. Iclarm, Los Ban Os. Laguna.

Froese, R., J. T. Thorson, and R. B. Reyes Jr. 2014. A Bayesian approach for estimating length-weight relationships in fishes. Journal of Applied Ichthyology 30:78–85.

Hoskonen, P., and J. Pirhonen. 2004. The effect of clove oil sedation on oxygen consumption of six temperate-zone fish species. Aquaculture Research 35:1002–1005.

Jackson, A. L., R. Inger, A. C. Parnell, and S. Bearhop. 2011. Comparing isotopic niche widths among and within communities: SIBER – Stable Isotope Bayesian Ellipses in R. Journal of Animal Ecology 80:595–602.

Jeannot, L.-L., J. P. Lozano-Peña, A. Zora, S. J. Brandl, and N. A. J. Graham. 2025. Seabird-derived nutrients influence feeding pathways and body size in cryptobenthic reef fishes. Proceedings of the Royal Society B: Biological Sciences 292:20250539.

McConnaughey, T., and C. P. McRoy. 1979. Food-Web structure and the fractionation of Carbon isotopes in the bering sea. Marine Biology 53:257–262.

McKinney, C. R., J. M. McCrea, S. Epstein, H. A. Allen, and H. C. Urey. 1950. Improvements in Mass Spectrometers for the Measurement of Small Differences in Isotope Abundance Ratios. Review of Scientific Instruments 21:724–730.

Morais, R. A., and D. R. Bellwood. 2018. Global drivers of reef fish growth. Fish and Fisheries 19:874–889.

Morais, R. A., and D. R. Bellwood. 2020. Principles for estimating fish productivity on coral reefs. Coral Reefs 39:1221–1231.

Oksanen, J., G. L. Simpson, F. G. Blanchet, R. Kindt, P. Legendre, P. R. Minchin, R. B. O'Hara, P. Solymos, M. H. H. Stevens, E. Szoecs, H. Wagner, M. Barbour, M. Bedward, B. Bolker, D. Borcard, G. Carvalho, M. Chirico, M. De Caceres, S. Durand, H. B. A. Evangelista, R. FitzJohn, M. Friendly, B. Furneaux, G. Hannigan, M. O. Hill, L. Lahti, D. McGlinn, M.-H. Ouellette, E. Ribeiro Cunha, T. Smith, A. Stier, C. J. F. Ter Braak, and J. Weedon. 2001, September 6. vegan: Community Ecology Package.

Ouellet, J.-F., J. Cabrol, È. Rioux, X. Bordeleau, and V. Lesage. 2024. Dealing with biases introduced by lipids in stable carbon and nitrogen isotope analyses: a solution based on 28 marine invertebrate, fish, and mammal species. Marine Ecology Progress Series 738:75–87.

Peterson, B. J., and B. Fry. 1987. Stable Isotopes in Ecosystem Studies. Annual Review of Ecology and Systematics 18:293–320.

Post, D. M. 2002. Using Stable Isotopes to Estimate Trophic Position: Models, Methods, and Assumptions. Ecology 83:703–718.

Post, D. M., C. A. Layman, D. A. Arrington, G. Takimoto, J. Quattrochi, and C. G. Montaña. 2007. Getting to the fat of the matter: models, methods and assumptions for dealing with lipids in stable isotope analyses. Oecologia 152:179–189.

R Core Team. 2025. R: A Language and Environment for Statistical Computing. R Foundation for Statistical Computing, Vienna, Austria.

Sandin, S. A., and I. Williams. 2010. Trophic Classifications of Reef Fishes from the Tropical U.S. Pacific (Version 1.0).

Sheppard, C. R. C., M. Ateweberhan, B. W. Bowen, P. Carr, C. A. Chen, C. Clubbe, M. T. Craig, R. Ebinghaus, J. Eble, N. Fitzsimmons, M. R. Gaither, C.-H. Gan, M. Gollock, N. Guzman, N. a. J. Graham, A. Harris, R. Jones, S. Keshavmurthy, H. Koldewey, C. G. Lundin, J. A. Mortimer, D. Obura, M. Pfeiffer, A. R. G. Price, S. Purkis, P. Raines, J. W. Readman, B. Riegl, A. Rogers, M. Schleyer, M. R. D. Seaward, A. L. S. Sheppard, J. Tamelander, J. R. Turner, S. Visram, C. Vogler, S. Vogt, H. Wolschke, J. M.-C. Yang, S.-Y. Yang, and C. Yesson. 2012. Reefs and islands of the Chagos Archipelago, Indian Ocean: why it is the world's largest no-take marine protected area. Aquatic Conservation: Marine and Freshwater Ecosystems 22:232–261.

Stier, A. C., and M. Leray. 2014. Predators alter community organization of coral reef cryptofauna and reduce abundance of coral mutualists. Coral Reefs 33:181–191.

Stock, B. C., A. L. Jackson, E. J. Ward, A. C. Parnell, D. L. Phillips, and B. X. Semmens. 2018. Analyzing mixing systems using a new generation of Bayesian tracer mixing models. PeerJ 6:e5096.
