# Supplementary material for: Contrasting cryptofaunal responses to seabird nutrient inputs illuminate coral reef productivity pathways
Source: Ecology. 2026 Jul 22;107(7):e70453. doi: 10.1002/ecy.70453 (PMC13390649; doi:10.1002/ecy.70453)
Supplement: Supplementary file 2 — Appendix S2. [file ECY-107-e70453-s001.pdf]

Appendix S2

Contrasting cryptofaunal responses to seabird nutrient inputs illuminate coral reef productivity pathways

Laura-Li Jeannot, Ruth E. Dunn, Joyce Velos, Gareth J. Williams, Cassandra E. Benkwitt, Nicholas A. J. Graham, Simon J. Brandl

Ecology

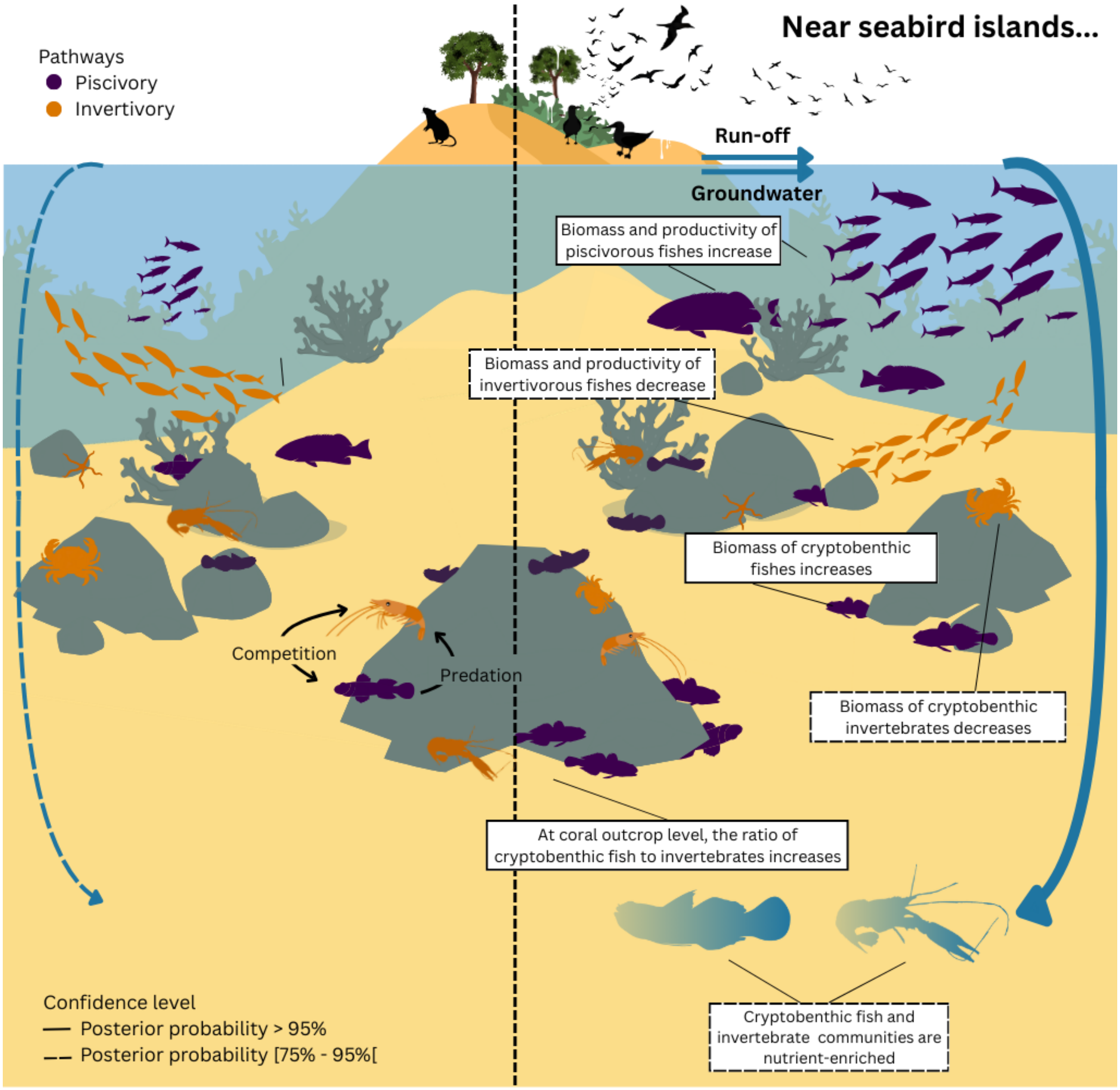

Figure S1: Seabird nutrients boost reef productivity via cryptofaunal pathways. Confidence level corresponds to posterior probabilities from fixed-effect hypothesis tests (Bürkner 2017). Figure created by Laura-Li Jeannot using icons of benthic organisms, terrestrial vegetation, rats and birds from Canva.

References

Bürkner, P.-C. 2017. brms: An R Package for Bayesian Multilevel Models Using Stan. Journal of Statistical Software 80:1–28.
